# TMS-Based Neurofeedback Training of Mental Finger Individuation Induces Neuroplastic Changes in the Sensorimotor System

**DOI:** 10.1523/JNEUROSCI.2189-24.2025

**Published:** 2025-07-24

**Authors:** Ingrid Angela Odermatt, Manuel Schulthess-Lutz, Ernest Mihelj, Paige Howell, Caroline Heimhofer, Roisin McMackin, Kathy Ruddy, Patrick Freund, Sanne Kikkert, Nicole Wenderoth

**Affiliations:** ^1^Neural Control of Movement Laboratory, Department of Health Sciences and Technology, ETH Zurich, Zurich 8092, Switzerland; ^2^Neuroscience Center Zurich (ZNZ), University of Zurich and ETH Zurich, Zurich 8057, Switzerland; ^3^Spinal Cord Injury Center, Balgrist University Hospital, University of Zurich, Zurich 8008, Switzerland; ^4^Discipline of Physiology, School of Medicine, Trinity Biomedical Sciences Institute, Trinity College Dublin, University of Dublin, Dublin D02 PN40, Ireland; ^5^School of Psychology, Queen’s University Belfast, Belfast BT9 5BN, United Kingdom; ^6^Wellcome Trust Centre for Neuroimaging, Queen Square Institute of Neurology, University College London, London WC1N 3AR, United Kingdom; ^7^Department of Neurophysics, Max Planck Institute for Human Cognitive and Brain Sciences, Leipzig D-04103, Germany; ^8^Future Health Technologies, Singapore-ETH Centre, Campus for Research Excellence and Technological Enterprise (CREATE), Singapore 138602, Singapore

**Keywords:** fMRI, finger representations, motor imagery, neurofeedback, plasticity, TMS

## Abstract

Neurofeedback (NF) training based on motor imagery is increasingly used in neurorehabilitation with the aim to improve motor functions. However, the neuroplastic changes underpinning these improvements are poorly understood. Here, we used mental “finger individuation,” i.e., the selective facilitation of single finger representations without producing overt movements, as a model to study neuroplasticity induced by NF. To enhance mental finger individuation, we used transcranial magnetic stimulation (TMS)-based NF training. During motor imagery of individual finger movements, healthy female and male human participants were provided visual feedback on the size of motor evoked potentials, reflecting their finger-specific corticospinal excitability. We found that TMS-NF improved the mental activation of finger-specific representations. First, intracortical inhibitory circuits in the primary motor cortex were tuned after training such that inhibition was selectively reduced for the finger that was mentally activated. Second, motor imagery finger representations in areas of the sensorimotor system assessed with functional MRI became more distinct after training. Together, our results indicate that the neural underpinnings of finger individuation, a well-known model system for neuroplasticity, can be modified using TMS-NF guided motor imagery training. These findings demonstrate that TMS-NF induces neuroplasticity in the sensorimotor system, highlighting the promise of TMS-NF on the recovery of fine motor function.

## Significance Statement

The activation of sensorimotor representations through motor imagery can be used to control brain–computer interfaces (BCIs) as assistive devices or training interventions. Here, we investigated how improved BCI control may change sensorimotor representations activated through motor imagery. We used BCI-neurofeedback based on TMS that allows for finger-specific feedback on corticospinal excitability. This training can be used to practice mental finger individuation, providing a model to study neuroplasticity. We demonstrate that motor imagery representations became more finger-specific after training, as evident in the tuning of intracortical inhibition and more distinct fMRI activation patterns in the sensorimotor system. These findings show that BCI training induces neuroplasticity in the sensorimotor system and shapes sensorimotor representations activated through motor imagery.

## Introduction

Body part representations in the primary somatosensory (S1) and motor (M1) cortices are activated when we execute movements and receive somatosensory inputs (Sanders et al., 2023). These representations can however also be activated without overt movement or somatosensory inputs, for example, by attempted movements of completely paralyzed (Kikkert et al., 2021; Guan et al., 2022) or amputated body parts (Kikkert et al., 2016; Bruurmijn et al., 2017; Wesselink et al., 2019), motor planning (Ariani et al., 2022), or motor imagery (Park et al., 2015; Pilgramm et al., 2016; Zabicki et al., 2017), i.e., the mere mental simulation of movements (Jeannerod, 1995). Such activation of sensorimotor representations can be used to control brain–computer interfaces (BCIs) that convert the elicited brain signals into control commands for external devices (e.g., a prosthetic arm) or into neurofeedback (referred to as BCI-NF). BCI-NF approaches allow users to gain volitional control of their brain activity and aim to induce neuroplastic changes in sensorimotor pathways (Sitaram et al., 2017; Colucci et al., 2022; Mane et al., 2023). Such BCI-NF training interventions are increasingly used in neurorehabilitation to aid motor recovery in the absence of overt motor output, mostly following a stroke (Ramos-Murguialday et al., 2013; Pichiorri et al., 2015; Niazi et al., 2022) or spinal cord injury (SCI; Donati et al., 2016). However, there is limited knowledge about the underlying neuroplastic changes of sensorimotor representations induced by BCI-NF (Sitaram et al., 2017; Bai et al., 2020; Simon et al., 2021).

Sensorimotor finger representations are well characterized and have been used to investigate neuroplasticity (Kolasinski et al., 2016b; Beukema et al., 2019; Wesselink et al., 2022). Thus, mental “finger individuation,” i.e., the selective facilitation of single finger muscles through motor imagery, may be a powerful model to study neuroplasticity induced by BCI-NF. Importantly, the hallmarks of finger representations can be assessed noninvasively using functional MRI (fMRI) and transcranial magnetic stimulation (TMS): First, finger representations in the primary sensorimotor cortex (SM1) are somatotopically organized, providing a point-to-point correspondence of individual fingers to a specific cortical area (Dechent and Frahm, 2003; Schweizer et al., 2008; Sanchez-Panchuelo et al., 2010; Besle et al., 2013; Kolasinski et al., 2016a; Huber et al., 2020; Sanders et al., 2023). Second, while finger representations are largely overlapping, the activity patterns associated with individual fingers are distinct in SM1 (Ejaz et al., 2015; Gooijers et al., 2022). Third, intracortical circuits regulate individual (imagined) finger movements through facilitating that specific finger representations while inhibiting the other fingers (Stinear and Byblow, 2003a,b, 2004; Sohn and Hallett, 2004; Neige et al., 2020).

**Figure 1.**
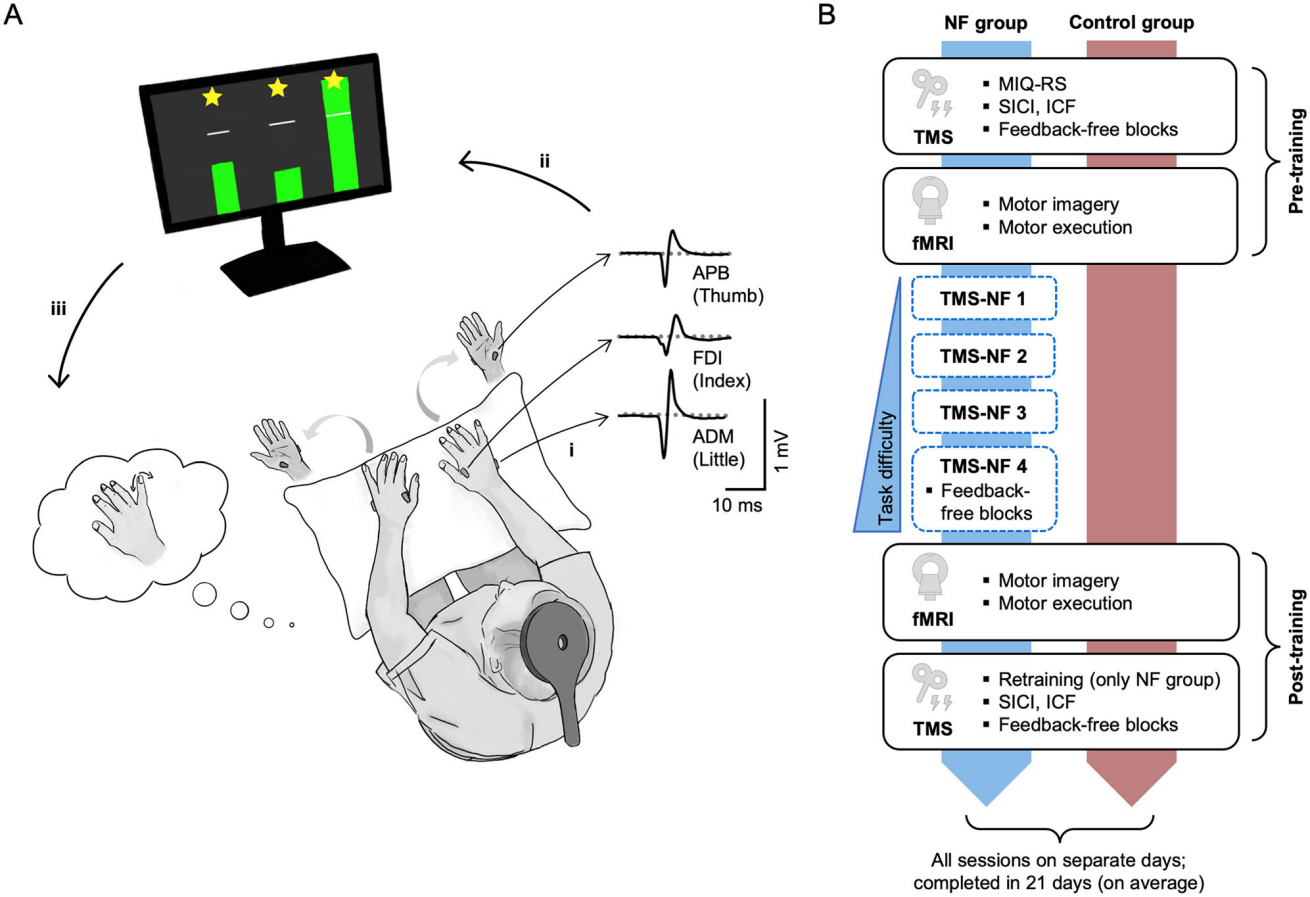
TMS-NF setup and study design. ***A***, TMS-NF setup. Participants imagined individual finger movements of the right hand (e.g., the little finger) while we recorded electromyography (EMG) of their finger muscles in both hands, i.e., left and right abductor pollicis brevis (APB), first dorsal interosseous (FDI), and abductor digiti minimi (ADM). ***i***, During motor imagery, we applied a TMS pulse with a round coil to elicit motor evoked potentials (MEPs) simultaneously in the three right hand finger muscles. ***ii***, We normalized the peak-to-peak amplitude of the MEPs to their baseline (based on preceding rest trials) and provided visual feedback on the normalized MEPs in the form of three bars (one for each finger muscle). The white lines indicate no change from baseline. If the bar of the instructed target finger was both above the white line and higher than the bars of the other two nontarget fingers, the trial was deemed successful (green bars). Unsuccessful trials were displayed with red bars (not depicted here). In a successful trial, participants could earn a star for each finger if the normalized MEP of the target finger was >1.5 and that of a nontarget finger <1. ***iii***, Participants used the visual feedback to adapt their motor imagery strategies. ***B***, Study design. The NF group (*n* = 16; blue) underwent four TMS-NF training sessions (TMS-NF 1–4) to train mental finger individuation. Task difficulty increased over sessions due to a transition from a blocked (i.e., one target finger per block) to an interleaved design (i.e., the target finger changed after each trial). The control group (*n* = 16; red) did not undergo any TMS-NF training. Both groups underwent identical pre- and post-training TMS and fMRI sessions. During the first pre-training TMS session, we assessed the kinaesthetic scale of the Movement Imagery Questionnaire (MIQ-RS). In the pre- and post-training TMS sessions, we assessed short-interval intracortical inhibition (SICI) and intracortical facilitation (ICF). We further tested motor imagery performance in feedback-free blocks that were identical to the TMS-NF training blocks that had an interleaved trial order, but with occluded feedback. For the NF group, feedback-free blocks were also assessed at the end of the fourth TMS-NF training session. A short retraining period of TMS-NF was added to the start of the post-training TMS session for the NF group. In the pre- and post-training fMRI sessions, we measured brain activity during individual finger motor imagery and during the execution of a paced finger-tapping task. Participants were instructed to kinaesthetically imagine individual finger movements in all sessions (see Extended Data Fig. 1-1 for verbatim instructions, Extended Data Fig. 1-2 for self-reported strategies in the TMS-NF training sessions, and Extended Data Fig. 1-3 for self-reported strategies in the fMRI pre- and post-training sessions).

We previously developed a BCI-NF approach based on TMS that enhances mental finger individuation (Mihelj et al., 2021). In this TMS-NF training, we provided visual feedback on motor evoked potentials (MEPs) of individual finger muscles (Fig. 1*A*). Here, we used TMS-NF as a specific type of BCI-NF training to guide motor imagery and ultimately induce neuroplasticity. First, we aimed to understand the effects of TMS-NF training on neurophysiological mechanisms. We therefore used paired-pulse TMS to probe short-interval intracortical inhibition (SICI), which measures postsynaptic GABA_A_-ergic inhibition (Ziemann et al., 1996; Fisher et al., 2002), and intracortical facilitation (ICF), considered to reflect glutamatergic facilitation (Liepert et al., 1998; Ziemann, 2013). By assessing SICI and ICF pre- and post-training, we tested whether TMS-NF training induces a release of intracortical inhibition and an increase of facilitation for the mentally activated finger. We then used fMRI and representational similarity analysis (RSA) to examine whether improved mental finger individuation through TMS-NF training is reflected in more distinct motor imagery finger representations after training. We further used a decoding analysis to investigate whether activity patterns elicited by motor imagery become more similar to those elicited by motor execution after TMS-NF training. Our main fMRI analysis focused on the SM1 hand cortex, as this brain area exhibits highly distinct finger representations (Ejaz et al., 2015; Ogawa et al., 2019). We further explored changes in motor imagery finger representations in secondary motor areas, i.e., the ventral (PMv) and dorsal premotor cortex (PMd), and the supplementary motor area (SMA), as these areas have been implicated in motor imagery (Hétu et al., 2013; Hardwick et al., 2018).

## Material and Methods

### Participants

For this study, we recruited 46 participants with the following inclusion criteria: no use of medication acting on the central nervous system, no neurological and psychiatric disorders, right-handed according to the Edinburgh Handedness Inventory (Oldfield, 1971), normal or corrected-to-normal vision, and no TMS (Wassermann, 1998; Rossi et al., 2021) and MRI contraindications. At the start of the study onset (i.e., at the beginning of the pre-training TMS session), we screened participants for their ability to perform kinaesthetic motor imagery using the kinaesthetic subscale of the Movement Imagery Questionnaire – Revised second version (MIQ-RS; Gregg et al., 2010; Thomschewski et al., 2017). In this questionnaire, participants are instructed to perform and then kinaesthetically imagine movements and rate this mental task from 1 (very hard to feel) to 7 (very easy to feel). We asked participants with low scores, i.e., more than 1 SD below the mean score reported in Gregg et al., (2010), whether they were able to mentally simulate the kinaesthetic experience of movements. If participants negated, we excluded them from the study.

We excluded a total of 14 participants after study enrolment due to the following: (1) reported difficulty to perform kinaesthetic motor imagery (2 participants); (2) a high resting motor threshold (RMT) that was above 80% of the maximum stimulator output (MSO) and resulted in difficulties to find a suitable testing intensity (6 participants); (3) reported discomfort during TMS or fMRI (3 participants), persistent background electromyography amplitude (bgEMG) that exceeded the online bgEMG control (>10 μV) during the first TMS session (1 participant); (4) excessive head motion in the first fMRI session, i.e., a mean displacement >1.1 mm (corresponding to half a voxel size) in the majority of runs (1 participant); or (5) being unsure about MRI contraindications (1 participant). Testing was completed by 16 participants in the NF group [age (mean ± SD): 25.1 ± 2.8 years; 8 females] and 16 participants in the control group (age: 26.4 ± 2.7 years; 8 females), adhering to the sample size calculation that was made prior to study onset [using G*Power v3.1, based on the effect size reported in Mihelj et al., (2021)]. The participants who completed testing did not report any major side effects after the TMS sessions. All research procedures were approved by the Cantonal Ethics Committee Zurich (BASEC Nr. 2018−01078) and were conducted in accordance with the declaration of Helsinki. All participants provided written informed consent prior to study onset.

### Experimental procedure

The NF group underwent four sessions of TMS-NF to train mental finger individuation. Additionally, we conducted pre- and post-training TMS and fMRI testing sessions to measure the neural consequences of TMS-NF (Fig. 1*B*). In the pre- and post-training TMS sessions, we used paired-pulse TMS protocols to quantify effects of TMS-NF on inhibition and facilitation in the primary motor cortex (M1) during motor imagery. In the pre- and post-training fMRI sessions, we acquired brain activity during imagined and executed individual finger movements to investigate neural finger representations. During the pre- and post-training TMS sessions, we additionally assessed motor imagery performance in feedback-free blocks, i.e., identical to TMS-NF, but with occluded feedback. We also assessed such feedback-free blocks at the end of the fourth (and last) TMS-NF session for the NF group. This allowed us to investigate the stability of motor imagery performance by comparing the measurement directly after TMS-NF training to the measurement in the post-training TMS session. Note that for the first three participants, we assessed the feedback-free blocks at the start of the first and the end of the fourth TMS-NF session rather than in the pre- and post-training TMS sessions. The control group did not receive any TMS-NF training but underwent identical pre- and post-training sessions as the NF group to control for test–retest effects. Importantly, we have already shown that a control group that received uninformative NF did not improve their ability to up- versus downregulate (finger-selective) modulation of MEPs (Ruddy et al., 2018; Mihelj et al., 2021). For one participant of the NF group, we repeated the post-training TMS session due to technical issues.

In the pre-training sessions, the NF and the control groups received identical, standardized instructions to imagine selective movements with the cued finger and were provided example strategies based on Mihelj et al. (2021) and Ruddy et al. (2018); see Extended Data Fig. 1-1 for verbatim instructions, and Extended Data Figs. 1-2 and 1-3 for self-reported strategies. For the post-training sessions, we instructed the NF group to apply the motor imagery strategies that they had acquired during the TMS-NF training.

We kept the experimenter and time of the day for the testing and training sessions consistent within each participant. All sessions took place on separate days, and the whole study was completed in an average of 21 d [NF group (mean ± SD): 19.5 ± 5.5; control group (mean ± SD): 21.7 ± 13.9].

### TMS and EMG setup

During the TMS sessions, participants sat in a comfortable chair with a headrest and placed their arms on a pillow on their lap. Surface EMG (Trigno Wireless, Delsys) was recorded from the left and right thumb (abductor pollicis brevis; APB), index finger (first dorsal interosseous; FDI), and little finger (abductor digiti minimi; ADM). EMG data were sampled at 1,926 Hz (National Instruments), amplified, and stored on a PC for offline analysis. For TMS-NF, a round coil with a 90 mm loop diameter was connected to a Magstim 200 stimulator (Magstim) to deliver single-pulse monophasic TMS. We used a round coil for TMS-NF to achieve a less focal stimulation. As such, we were able to elicit motor evoked potentials (MEPs) in all three measured finger muscles of the right hand in the same coil position as in the setup of Mihelj et al. (2021). For paired-pulse TMS protocols, a 70 mm figure-of-eight coil was connected to two coupled Magstim stimulators. Here, we used a coil which allows for a more focal stimulation and optimally target the M1 representation of the right FDI. All stimuli were provided using custom MATLAB scripts (MATLAB 2020b, MathWorks) and Psychophysics Toolbox-3 (Brainard, 1997; Kleiner et al., 2007).

### TMS-based neurofeedback task

We used similar procedures as in Mihelj et al. (2021) to train participants to selectively modulate their corticospinal excitability through motor imagery using TMS-NF. A TMS-NF trial started with a preparatory rest period of 1–2 s. During this time, the bgEMG of all measured finger muscles on the left and right hand was computed as the root mean square (rms) of the EMG signal within a sliding window of 100 ms. Participants saw six dots on the screen, representing the bgEMG of the individual muscles. The dots were green when the bgEMG was <10 μV and turned red otherwise. The aim of this bgEMG control was to prevent participants from making subtle movements or muscle contractions. Only when the bgEMG in all muscles was <10 μV for a minimum of 1 s did the trial proceed to the motor imagery (or rest) period. During this period a visual cue appeared on the screen that instructed the participant to perform finger-selective motor imagery of the right hand (“thumb,” “index,” or “little”) or to rest (“rest”). The first 10 trials in each block were rest trials, which we collected to determine a baseline for each finger muscle. The motor imagery (or rest) period of a trial lasted for a jittered period of 4–6 s to avoid anticipation effects for the TMS pulse (Tran et al., 2021). If the bgEMG control indicated that the bgEMG rms exceeded 10 μV in any muscle, the motor imagery (or rest) period restarted. The bgEMG control only stopped in the last 0.5 s before the TMS pulse was applied and the dots remained green, regardless of the bgEMG values. After each TMS pulse, we computed the MEP peak-to-peak amplitudes of the three right hand finger muscles. The feedback (or fixation cross for rest trials) was displayed 1 s after the TMS pulse and lasted for 3 s. The feedback (Fig. 1*A*) was based on the normalized MEP amplitudes, which were computed by dividing the peak-to-peak MEP amplitude of a finger muscle by the median of the nine rest MEP amplitudes of the same finger muscle in the corresponding block (the first rest trial was discarded). These normalized MEPs were displayed as three bars representing the thumb, index, and little finger MEPs, respectively. The white lines represented the baseline MEPs of the three finger muscles. If the bar exceeded the white line, the normalized MEP of the cued target finger was >1, i.e., the current MEP was higher than the baseline MEP, indicating facilitation. If the bar was below the white line, the current MEP was below the baseline MEP (normalized MEP < 1), indicating suppression. If the normalized MEP of the cued target finger was both >1 and higher than the normalized MEPs of the other two (nontarget) finger muscles, the trial was deemed successful, and the bars were displayed in green. If not, the trial was deemed unsuccessful, and the bars were displayed in red. In a successful trial, participants could additionally reach up to three stars, one for each finger. To reach a star for the cued finger, the normalized MEP had to be >150% of the other two nontarget fingers. For the nontarget fingers, the normalized MEPs had to be <1.

### TMS-based neurofeedback training sessions

For the TMS-NF training sessions, we positioned the round coil over the vertex oriented to induce a posterior-anterior current flow in the left M1. We first determined a stimulation intensity that elicited MEPs in all three finger muscles of the right hand. These MEPs should be in a range from which participants could up- and downregulate using motor imagery strategies, defined as 115% of the RMT of all three fingers. We therefore first measured the RMT as the minimum intensity needed to elicit MEPs of 50 μV amplitude with a probability of 0.5 (Rossini et al., 1994) in all three finger muscles simultaneously at rest. To do so, we used adaptive threshold hunting based on maximum likelihood parameter estimation by sequential testing (PEST; Awiszus, 2003), which was shown to be a highly reliable method to estimate the RMT (Sen et al., 2017; Dissanayaka et al., 2018). PEST uses a probabilistic method to estimate the minimum TMS test stimulus (TS) intensity needed to elicit MEPs of a defined amplitude, here 50 μV for the RMT, in 50% of trials. We used an automated PEST script, implemented in MATLAB (Calvert et al., 2020), that incorporates the PEST function from the MTAT2.0 program (Awiszus and Borckardt, 2011) as described in McMackin et al. (2024). The peak-to-peak amplitude of the MEP of the targeted muscle is calculated online (here the lowest of the three MEPs) and passed to the algorithm following pulse delivery. PEST then recommends a TS intensity for the following trial, which is more likely to be the RMT, and was automatically adjusted by a microcontroller. This procedure was repeated for 20 trials to converge with sufficient confidence on an estimate of the RMT (Dissanayaka et al., 2018). As MEP amplitudes in the first trial are typically higher because of the novelty of the TMS sensation, we repeated the first trial, resulting in 21 trials for each block of adaptive threshold hunting. To ensure that the MEPs were not influenced by bgEMG, a trial was repeated automatically if the rms amplitude exceeded 10 μV in any of the three right hand finger muscles. The experimenter visually controlled for a reliable convergence of the TS, i.e., a probability of ∼0.5 to elicit MEPs of the defined amplitude in the last trials and otherwise repeated the RMT measure.

Next, we tested the estimated stimulation intensity for TMS-NF of 115% RMT and adjusted the intensity and/or the coil position if it did not elicit MEPs in all three finger muscles in each trial or if it resulted in ceiling effects in any of the three finger muscles. We then provided six blocks of TMS-NF in each training session. Each block consisted of 10 rest trials and 24 motor imagery trials, followed by a short break of 30 s between the blocks and a longer break after every second block. If the experimenter identified changes in corticospinal excitability based on MEP amplitudes during a session, the testing intensity was adjusted between blocks with longer breaks. During the first session, TMS-NF consisted of a blocked design, i.e., we cued a single finger for two consecutive blocks. This allowed participants to explore different motor imagery strategies. In the second session, we reduced the number of repetitions per finger to eight trials and to four in the third session. The order of the blocks and cued fingers was pseudorandomized and balanced across participants. In the fourth session, the trial order was completely interleaved and counterbalanced across cued fingers. We aimed for an interleaved order of trials as beneficial effects of such interleaved practice on delayed recall and long-term retention have been shown (Wright et al., 2016). Due to the required change of the motor imagery strategies after each trial, difficulty increases with an interleaved order. As Mihelj et al. (2021) showed a high increase of performance in a blocked trial order in TMS-NF, we designed a gradual change from a blocked to an interleaved order over the four sessions in this study. At the end of each session, participants noted down the strategies they had used for each of the fingers (Extended Data Fig. 1-2) and rated each strategy on a scale from 1 (not successful at all) to 7 (very successful).

For the NF group, the post-training TMS session started with a short retraining consisting of two blocks of TMS-NF with four repetitions per finger.

For the feedback-free measures, we assessed two blocks that were identical to TMS-NF with an interleaved trial order, except that no visual feedback was provided. Instead, a white fixation cross appeared on the screen for the same duration (3 s).

### Offline EMG data processing

**Figure 2.**
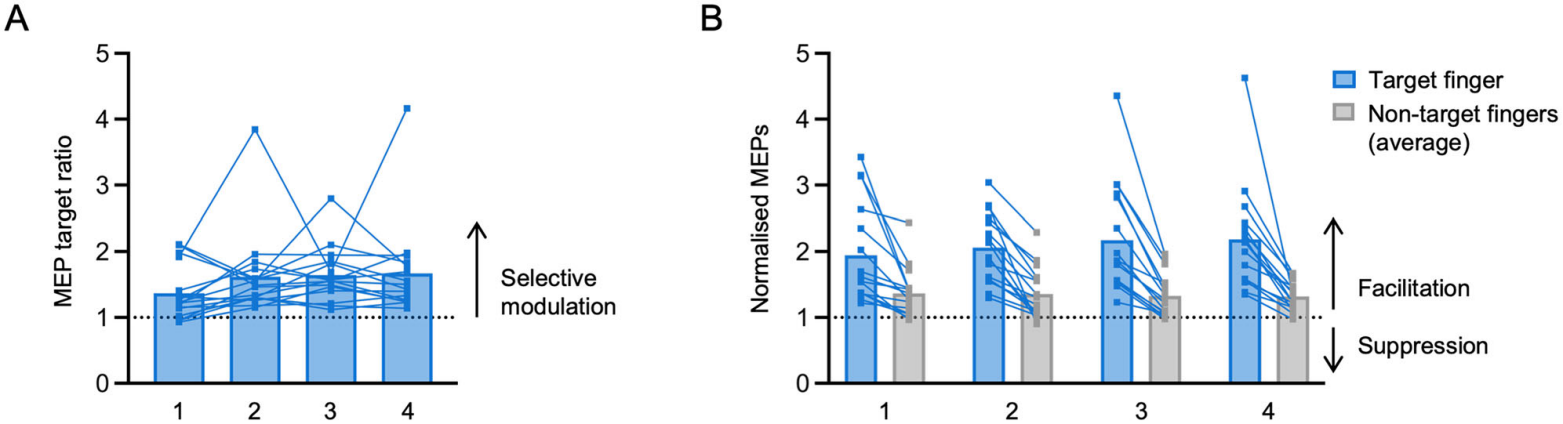
Motor imagery in TMS-NF training sessions. The *x*-axis corresponds to the four TMS-NF training sessions. Note that no statistics were applied on the training data as the motor imagery performance is confounded by increased difficulty through the transition from a blocked to an interleaved design. ***A***, MEP target ratio, i.e., the ratio between the normalized MEP (to the baseline at rest) of the target finger and the larger normalized MEP of the two nontarget fingers. Values >1 indicate a finger-selective modulation of corticospinal excitability. ***B***, Normalized MEPs of the target fingers (blue) and the average normalized MEPs of the two nontarget fingers (gray). Squares depict data of individual participants.

**Figure 3.**
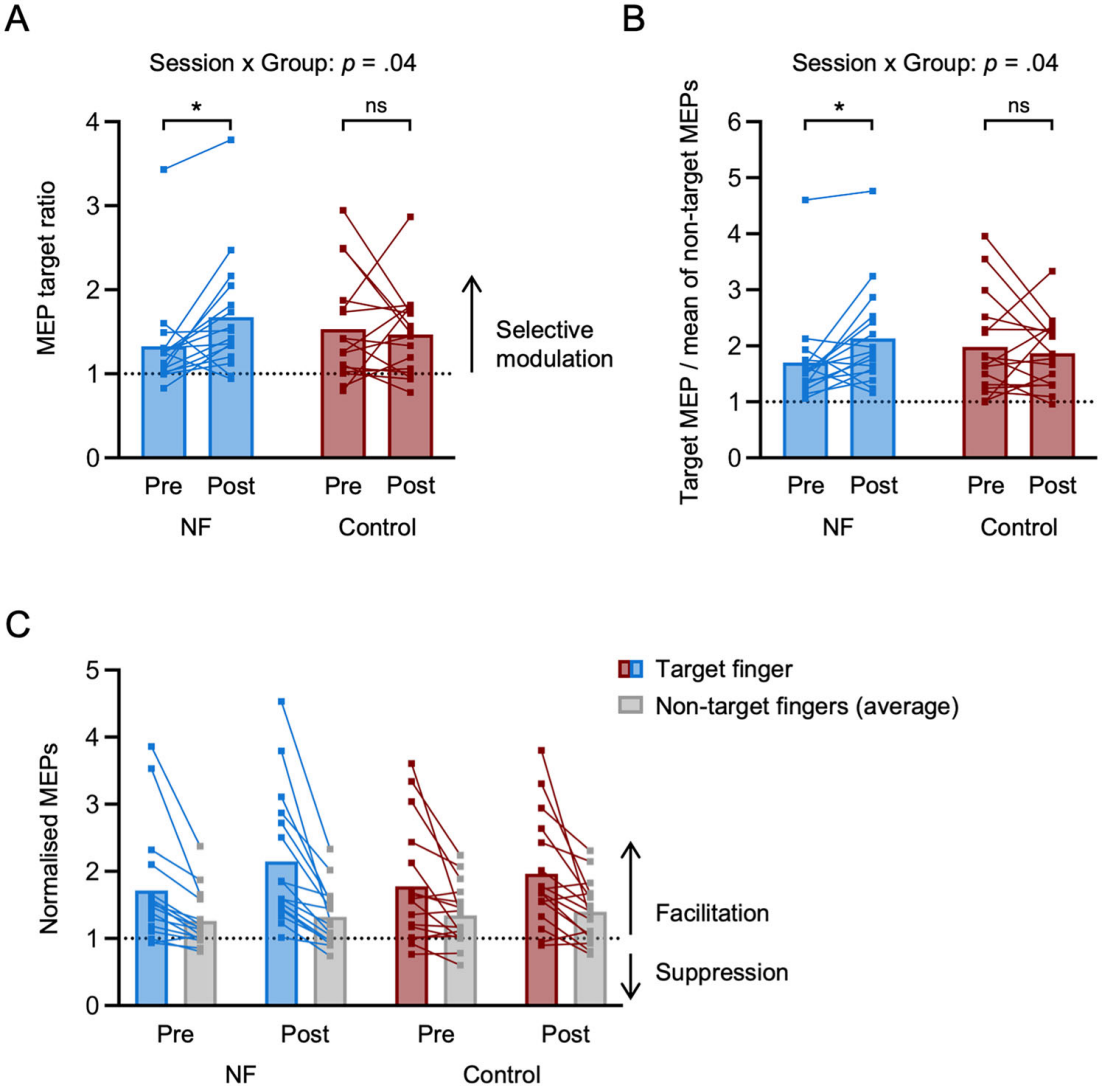
Motor imagery performance improves from pre to post TMS-NF training. ***A***, MEP target ratio, i.e., the ratio between the normalized MEP (to the baseline at rest) of the target finger and the larger normalized MEP of the two nontarget fingers. Values >1 indicate a finger-selective modulation of corticospinal excitability. The data depicted corresponds to the feedback-free blocks acquired in the TMS pre- and post-training testing sessions for the NF (blue) and control (red) groups. One participant in the NF group was classified as an outlier based on the pre-training session. Removing the data of this participant did not change the interpretation of the results (session by group interaction: *F*_(1,30.1)_ = 4.13, *p* = 0.05, Cohen's *d* = 0.74, 95% CI for Cohen's *d*: [0.00, 1.47]; NF pre- to post-training: *t*_(29.2)_ = −2.38, *p* = 0.02, Cohen's *d* = 0.89, 95% CI for Cohen's *d*: [0.12, 1.66]; control pre- to post-training: (*t*_(28.9)_ = 0.51, *p* = 0.62, Cohen's *d* = 0.18, 95% CI for Cohen's *d*: [−0.54, 0.90])*. **B***, Control analysis accounting for changes in MEPs in all measured finger muscles*.* We repeated the analysis reported in ***A***, using the ratio between the normalized MEP of the target finger muscle and the mean of the normalized MEPs of the two nontarget finger muscles, as depicted in ***C***. As in ***A***, the NF group improved motor imagery performance from pre- to post-training (*t*_(29.7)_ = −2.44, *p* = 0.02, Cohen's *d* = 0.88, 95% CI for Cohen's *d*: [0.13, 1.63]), whereas the control group did not (*t*_(29.6)_ = 0.73, *p* = 0.47, Cohen's *d* = 0.26, 95% CI for Cohen's *d*: [−0.47, 0.99]; significant session by group interaction: *F*_(1,30.1)_ = 4.84, *p* = 0.04, Cohen's *d* = 0.80, 95% CI for Cohen's *d*: [0.05, 1.54]). The covariate of the ratio between the normalized bgEMG and the mean of the normalized bgEMG of the two nontarget fingers did not reach significance (*F*_(1,47.4)_ = 0.38, *p* = 0.54, Cohen's *d* = 0.18, 95% CI for Cohen's *d*: [−0.39, 0.75]). Excluding the outlier participant in the NF group did not change significance of the results (session by group interaction: *F*_(1,29.5)_ = 4.91, *p* = 0.03, Cohen's *d* = 0.82, 95% CI for Cohen's *d*: [0.06, 1.56]; NF pre- to post-training: *t*_(28.5)_ = −2.42, *p* = 0.02, Cohen's *d* = 0.89, 95% CI for Cohen's *d*: [0.13, 1.65]; control pre- to post-training: *t*_(28.9)_ = 0.73, *p* = 0.47, Cohen's *d* = 0.26, 95% CI for Cohen's *d*: [−0.46, 0.99]). ***C***, Normalized MEPs of the target fingers and the averaged normalized MEP of the two nontarget fingers (gray). Squares depict data of individual participants. *p < 0.05; ns, nonsignificant.

Preprocessing of EMG data was performed using custom Python 3.7 scripts. EMG data from all six hand muscles were bandpass filtered (30−800 Hz) separately for the 5–105 ms of bgEMG before the TMS pulse was applied and for the 15–60 ms after the pulse that contained the MEP to avoid smearing of the MEP into the bgEMG. An additional 50 Hz notch filter was applied to the bgEMG data only. We calculated the rms of the bgEMG, calculated the peak-to-peak MEP amplitude, and normalized the MEP and bgEMG of each motor imagery trial and finger muscle by the baseline of the rest trials in the corresponding TMS-NF block. We then split the dataset into training (NF 1–4; Fig. 2) and feedback-free data (Fig. 3). Note that during TMS-NF, no online filters were applied. For all statistical analyses we used the feedback-free blocks from the pre- and post-training TMS sessions. For the three participants in the NF group who did not perform the feedback-free blocks in the post-training TMS session, we took the data from the feedback-free blocks in the fourth TMS-NF training session instead. This choice was motivated by a control analysis using the data from the remaining 13 participants, which showed that the MEP target ratio differed only insignificantly between the fourth TMS-NF session and the post-training TMS session (classical Wilcoxon signed-rank test: *W* = 46, *p* = 1.00; Bayesian Wilcoxon signed-rank test: BF_10_ = 0.29, indicating moderate evidence for no differences across the two measurement time points).

During offline analysis, we excluded all trials in which the rms amplitude of any of the muscles exceeded 7 µV (2.80% of total feedback-free trials). We further excluded trials with rms values that were 2.5 SD above or below the mean bgEMG of each muscle (10.55% of total feedback-free trials). Separately for both groups and sessions, we excluded in total the following % of trials: NF pre-training (mean [min–max]: 10.68% [2.08–20.83%]; NF post-training: 14.84% [4.17–29.17%]; control pre-training: 14.19% [6.25–31.25%]; control post-training: 13.67% [2.08–29.17%]). Using the remaining trials, we quantified motor imagery performance, following similar procedures as in Mihelj et al. (2021). We calculated the MEP target ratio as the ratio between the normalized MEP of the cued target finger muscle and the higher of the nontarget MEPs. An MEP target ratio >1 indicates a finger-selective upregulation of corticospinal excitability; a value of 1 reflects no modulation; and values <1 show a finger-selective downregulation. We then averaged the resulting MEP target ratio across all trials per participant and per session. We additionally computed the bgEMG target ratio using the bgEMG instead of MEPs and added it as a covariate in the linear mixed-effects model to control for subtle selective muscle contractions (bgEMG rms <7 µV) in the motor imagery period. To confirm that disregarding the lower of the two normalized nontarget MEPs did not affect the results, we additionally calculated the ratio of the normalized MEP of the target finger muscle and the average of the normalized MEPs of both nontarget finger muscles.

### Paired-pulse TMS measurements and analysis

We used adaptive threshold hunting to assess short-interval intracortical inhibition (SICI), intracortical facilitation (ICF), and a single pulse (nonconditioned) protocol in the right FDI (i.e., index finger) while participants imagined moving either their index finger or while they imagined moving their thumb. This resulted in two motor imagery conditions where the index finger was either the target or a nontarget finger.

We positioned the figure-of-eight-coil over the hotspot of the right FDI, i.e., the coil location eliciting the highest and most consistent MEPs in the right FDI. The coil was held tangential to the scalp at a 45° angle to the mid-sagittal line to achieve a posterior-anterior direction of current flow in the brain. This optimal coil location was registered in the neuronavigation software (Brainsight Frameless, Rogue Research). The position of the coil and the participant's head were monitored in real-time using the Polaris Vicra Optical Tracking System (Northern Digital). First, we determined the RMT of the right FDI using adaptive threshold hunting (as described in TMS-based neurofeedback training sessions). Next, we measured the maximum MEP: We applied 10 pulses where the intensity of the first pulse was set to 50% of MSO, followed by three repetitions of 65, 80, and 95% of the MSO. The first trial was discarded because of the novelty of TMS sensation, and the maximum MEP was defined as the largest of the nine remaining MEPs without outliers (NF pre-training (mean [min–max]): 2.40 mV [0.69–5.69 mV]; NF post-training: 2.92 mV [0.82–9.97 mV]; control pre-training: 3.12 mV [0.58–7.18 mV]; control post-training: 3.86 mV [1.34–9.97 mV]).

For SICI and ICF we set the conditioning stimulus (CS) intensity to 70% RMT. The inter-stimulus interval (ISI) was set at 2 ms for SICI (Peurala et al., 2008; Ruddy et al., 2018) and 12 ms for ICF. In each block, we measured the TS during motor imagery which had a 50% probability of evoking an MEP of >50% of the maximum MEP as target MEP. We tested one protocol per block, and two separate PEST protocols ran in an interleaved manner within a block to track the two TS of the motor imagery conditions (i.e., imagined index finger or imagined thumb movements) with 20 trials each. We determined the TS for both motor imagery conditions in the same block to control for changes in corticospinal excitability throughout the session. The cued finger (i.e., index or thumb) was repeated four times each. The structure of a trial was consistent with TMS-NF, except that a fixation cross and no feedback was presented for 2 s after applying the TMS pulse(s). We applied a similar online bgEMG control as in TMS-NF; however, as we focused on motor imagery of the right index finger and thumb, the trial only paused when the bgEMG of the right FDI or APB exceeded 10 μV. For the other finger muscles, the dots representing the bgEMG turned yellow instead of red if bgEMG exceeded 10 μV and the trial proceeded normally. Participants were instructed to relax their muscles if a dot turned yellow but to primarily focus on motor imagery. If the bgEMG in the right APB or FDI exceeded 10 μV in the 5–105 ms before the CS (or TS in the single pulse protocol), the trial was repeated automatically. The order of stimulation protocols and which motor imagery condition was presented first in a block was balanced across participants and kept consistent for the pre- and post-training sessions within participant. The second assessed protocol was always the single pulse protocol. If the threshold of one of the two motor imagery conditions did not converge reliably, the block was repeated (see Extended Data Fig. 4-1 for number of repetitions per participant). For repeated blocks, the plots of stimulation intensities and trials that showed positive and negative responses for each tested intensity were visually inspected by the experimenter and an independent, blinded researcher after the session to decide which of the repetitions was used for further analysis: If possible, the thresholds for both motor imagery conditions (target vs nontarget) were taken from the same block, unless the threshold of one motor imagery condition clearly converged better in another block.

We expressed inhibition (and facilitation) as the % change in TS intensity in the SICI (or ICF) protocol compared with the single pulse protocol. For inhibition, positive values indicate that a higher intensity was needed to elicit MEP amplitudes of at least 50% of the maximum MEP in the SICI compared with the single pulse protocol. For facilitation, positive values indicate that the ICF protocol resulted in a lower intensity than the single pulse protocol to elicit an MEP amplitude of at least 50% of the maximum MEP:

$$\mathrm{Inhibition}\;\text{\%}=\;\frac{\mathrm{TS}(\mathrm{SICI})-\mathrm{TS}(\mathrm{single}\;\mathrm{pulse})}{\mathrm{TS}(\mathrm{single}\;\mathrm{pulse})}\;\times \;100,$$

$$\mathrm{Facilitation}\;\text{\%}=\;\frac{\mathrm{TS}(\mathrm{ICF})-\mathrm{TS}(\mathrm{single}\;\mathrm{pulse})}{\mathrm{TS}(\mathrm{single}\;\mathrm{pulse})}\;\times \;(-100).$$

We then computed the pre- to post-training differences in inhibition, or facilitation, for the two motor imagery conditions.

### fMRI tasks

We employed two paradigms in the pre- and post-training fMRI sessions to uncover neural changes after TMS-NF training. First, we assessed brain activity during individual finger motor imagery to analyze how finger-specific activity patterns change after TMS-NF training. Second, to compare these motor imagery activity patterns to those of motor execution, we additionally assessed motor execution in a paced finger-tapping task. Participants viewed a fixation cross centered on a screen through a mirror mounted to the head coil. For the motor imagery runs, participants were visually cued by the words “thumb,” “index,” “little,” or “rest.” Each motor imagery period was followed by a jittered rest period of 3–4 s during which a fixation cross was displayed instead of the task instruction. To ensure that participants did not execute any finger movements during this task, an experimenter visually controlled for finger movements inside the scanner room. If any movements were detected, we stopped the run, instructed the participant to refrain from executing finger movements, and repeated the run. We acquired four motor imagery runs using a blocked paradigm with block lengths of 7.5 s. In every run, each of the three fingers and rest were cued 12 times in a counterbalanced order, resulting in 48 trials per condition and session. Each motor imagery run lasted for 9 min 8 s.

During the motor execution runs, the participants' right index, ring, middle, and little fingers were placed on the buttons of a four-button response box, with the thumb placed on the side of the box. Participants viewed a fixation cross. They were then visually cued by the words “thumb,” “index,” “middle,” “ring,” “little,” or “rest” appearing above the fixation cross to perform paced button presses with the corresponding finger (or to tap the side of the button box with the thumb) or to rest. The fixation cross blinked at 1.4 Hz to instruct the pace. In the rest condition, no fixation cross was displayed. We acquired six motor execution runs using a blocked paradigm with block lengths of 7.5 s. No breaks were provided between trials. In every run, each of the five fingers and rest were presented five times in a counterbalanced order, resulting in 30 trials per condition and session. The number of trials is comparable with other studies investigating finger representations with motor execution paradigms and multivariate pattern analysis (MVPA; Shen et al., 2014; Ogawa et al., 2019; Arbuckle et al., 2020; Kikkert et al., 2021; Sanders et al., 2023). Each motor execution run lasted for 4 min 5 s.

### fMRI data acquisition

We used a 3 T Siemens Magnetom Prisma scanner with a 64-channel head-neck coil (Siemens Healthcare) to acquire fMRI data. For the anatomical T1-weighted images, we used a Magnetization Prepared Rapid Gradient Echo (MPRAGE) protocol with the following acquisition parameters: 160 sagittal slices; resolution, 1 × 1.1 × 1 mm^3^; field of view (FOV), 240 × 240 × 160 mm; repetition time (TR), 2,300 ms; echo time (TE), 2.25 ms; flip angle, 8°. For the task-fMRI data acquisition, we used an echoplanar imaging (EPI) sequence covering the whole brain and the cerebellum with the following acquisition parameters: 66 transversal slices; resolution, 2.2 mm^3^ isotropic; FOV, 210 × 210 × 145 mm^3^; TR, 846 ms; TE, 30 ms; flip angle, 56°; acceleration factor, 6; and echo spacing, 0.6 ms. We acquired 636 and 278 volumes for each of the motor imagery and motor execution runs, respectively. To measure B0 deviations we used a fieldmap with the same resolution and slice angle as the EPI sequence and the following acquisition parameters: TR, 649 ms; TE1, 4.92 ms; TE2, 7.38 ms.

### fMRI data preprocessing and coregistration

DICOM images were converted to nifti format using MRIcroGL v13.6 (https://www.nitrc.org/projects/mricrogl). MRI analysis was conducted using tools from FSL v.5.0.7 (http://fsl.fmrib.ox.ac.uk/fsl) unless stated otherwise. The following preprocessing steps were applied to the fMRI data using FSL's Expert Analysis Tool (FEAT): motion correction using MCFLIRT (Jenkinson et al., 2002), brain extraction using the automated brain extraction tool (BET; Smith, 2002), spatial smoothing using a 3 mm full-width at half-maximum (FWHM) Gaussian kernel, and high-pass temporal filtering with a 100 s cutoff. Nonbrain tissue from the T1-weighted images of the pre- and post-training fMRI session was removed using BET and/or Advanced Normalization Tools (ANTs) v2.3.5 (http://stnava.github.io/ANTs) to receive a binarized mask of the extracted brain. Image coregistration was performed in separate, visually inspected steps. For each participant, we created a mid-space, i.e., an average space, between the T1-weighted images and its binarized brain masks of the pre- and the post-training sessions. We then used the mid-space brain mask to brain extract the mid-space T1-weighted image. By using this T1-weighted mid-space for coregistration, we ensured that the extent of reorientation required in the registration from functional to structural data was equal in the pre- and post-training fMRI sessions. Functional data were then aligned to the brain-extracted T1-weighted mid-space, initially using six degrees of freedom and the mutual information cost function, and then optimized using boundary-based registration (BBR; Greve and Fischl, 2009). To correct for B0 distortions, a fieldmap was constructed for B0 unwarping and added to the registration. For one participant, the fieldmap worsened coregistration in the MRI pre-session and was therefore not applied. Three participants were taken out of the scanner for a brief break during the MRI pre-training session, and the fieldmaps were only applied to the functional runs that were acquired with the same head position as the fieldmap. Structural images were transformed to Montreal Neurological Institute (MNI-152) standard space by nonlinear registration (FNIRT) with 12 degrees of freedom. The resulting warp fields were then applied to the functional statistical images.

Each functional run was assessed for excessive motion and excluded from further analyses if the absolute mean displacement was greater than half the voxel size (i.e., >1.1 mm). This resulted in the exclusion of one motor execution fMRI run for two participants of the NF group.

### Univariate analysis

To assess univariate task-related activity of motor imagery and execution, time series statistical analysis was carried out per run using FMRIB's Improved Linear Model (FILM) with local autocorrelation, as implemented in FEAT. We defined one regressor of interest for each individual finger and obtained activity estimates using a general linear model (GLM) based on the gamma hemodynamic response function (HRF) and the temporal derivatives. We added nuisance regressors for the six motion parameters (rotation and translation along the *x*, *y*, and *z*-axis) and white matter (WM) and cerebrospinal fluid (CSF) time series. We further assessed the data for excessive head motion and scrubbed volumes with an estimated absolute displacement >1.1 mm (i.e., half of the functional voxel size; maximum percentage of volumes scrubbed in a run = 2.16%).

For motor execution, we carefully inspected which finger participants used to press the button during each trial by examining the recorded button presses. When needed, we adjusted the finger movement regressors: If the button of a noninstructed finger was pressed during a motor execution trial, then we adjusted the regressors such that the trial was assigned to this noninstructed, moving, finger. If a button press indicated that the switch to the next cued finger was made with a delay, then we adjusted the corresponding block length and the movement onset of the next trial. To test whether these adjustments resulted in considerable differences in the number of trials across fingers, we conducted a Friedman test with the factor finger (thumb, index, little finger) separately for the two groups. We found no significant main effect for the control group ( $\chi_{2}^{2}=3.44,$ *p* = 0.18) and a trend for a main effect for the NF group ( $\chi_{2}^{2}=5.24,$ *p* = 0.07). Post hoc Conover comparisons for the NF group did not reveal any significant difference in the number of trials for any finger pair (all *p_(FDR)_* ≥ 0.10). In the NF group, the number of index finger trials (mean ± SD: 59.63 ± 1.67) was on average less than 0.5 trials higher than the number of thumb trials (59.19 ± 1.72) and little finger trials (59.25 ± 1.91).

For motor imagery, we defined contrasts for each finger > rest, and overall task-related activity by contrasting all finger conditions > rest. We then averaged across runs at the individual participant level using fixed effect analysis. To define the motor imagery network, we entered the overall activity > rest contrast of the pre-training fMRI session of all participants (across the NF and control groups) into a mixed-effects higher-level analysis and thresholded it at *Z* > 3.1, *p*_FWE_ < 0.05 at cluster level. Next, we aimed to test for activity changes from pre- to post-training and whether that differed between the groups. To do so, we defined pre > post and post > pre contrasts for the overall task-related activity at the individual participant level. We then used a mixed effect GLM to test for the group difference in a two-sample unpaired *t* test. Additionally, to investigate group-specific effects in the pre- to post-training changes, we used mixed effect GLMs to compute one-sample *t* tests on the pre > post and post > pre contrasts. Next, we investigated whether changes in the overall task-related activity were associated with changes in motor imagery performance (i.e., the MEP target ratio). To do so, we entered the pre- to post-training contrasts and the demeaned MEP target ratio changes in a mixed effect GLM to test the interaction effect, i.e., whether group differences in the pre- to post-training contrast maps vary as a function of motor imagery performance changes.

### Definition of regions of interest

Similar to previous studies using MVPA to investigate finger representations (Kikkert et al., 2016, 2021; Kolasinski et al., 2016b; Beukema et al., 2019; Wesselink et al., 2022; Rabe et al., 2023) and imagined hand actions (Park et al., 2015; Pilgramm et al., 2016; Zabicki et al., 2017), we defined anatomical regions of interest (ROIs). These ROIs were based on the probabilistic Brodmann area (BA) parcellation using FreeSurfer v6.0 (https://surfer.nmr.mgh.harvard.edu/; Dale et al., 1999; Fischl et al., 1999; Fischl, 2012). We reconstructed the cortical surface of each individual participant's T1-weighted mid-space image. We created a primary sensorimotor hand area ROI using similar procedures as in Kikkert et al. (2021, 2023). We first transformed BAs 1, 2, 3a, 3b, 4a, and 4p to volumetric space, merged them into an SM1 ROI, and filled any holes. Next, we nonlinearly transformed axial slices spanning 2 cm medial/lateral to the anatomical hand knob (Yousry et al., 1997) on the 2 mm MNI standard brain (min–max MNI *z*-coordinates = 40–62) to each participant's native structural space. Lastly, we used this mask to restrict the SM1 ROI and extracted an SM1 hand area ROI.

We further defined ROIs for dorsal and ventral premotor cortex (PMd and PMv), and supplementary motor area (SMA) by masking BA6 with the corresponding areas of the Human Motor Area Template (HMAT) atlas (Mayka et al., 2006) that were transformed into native space. For these masks, we then subtracted any overlap, as well as overlap with the SM1 hand area to avoid a voxel being assigned to multiple ROIs.

### Representational similarity analysis

While univariate analysis shows clusters of enhanced activity during imagined or executed finger movements, MVPA allows to investigate the fine-grained finger-specific activity patterns. Here, we used RSA to test the inter-finger distances of voxel-wise activity patterns elicited by individual finger motor imagery. We aimed to see whether these imagined finger movement representations became more distinct after TMS-NF training. To do so, we used the RSA toolbox (Nili et al., 2014) and MATLAB R2015a. We computed the distance between the activity patterns for each finger pair in the SM1 hand ROI, SMA, PMd, and PMv using the cross-validated Mahalanobis distance, also called crossnobis distance (Nili et al., 2014). Specifically, we extracted the voxel-wise parameter estimates (betas) for motor imagery of each finger > rest per run and the model fit residuals under an ROI. These extracted betas were then pre-whitened using the model fit residuals. To calculate the crossnobis distance for each finger pair, we used the four motor imagery runs as independent cross-validation folds and averaged the resulting distances across the folds. If it is impossible to statistically differentiate between motor imagery conditions (i.e., when this parameter is not represented in the ROI), the expected value of the distance estimate would be 0. If it is possible to distinguish between activity patterns, this value will be larger than 0 (Yokoi et al., 2018). We estimated the strength of the finger representation or average inter-finger distances in each ROI as the average distance of all finger pairs. Average inter-finger distances larger than 0 indicates that there is neural information content in the ROI that can statistically differentiate between motor imagery of individual fingers.

### Cross-task classification

Next, we aimed to investigate whether neural activity patterns elicited by individual finger motor imagery became more similar to those observed during motor execution following TMS-NF training. To do so, we performed a cross-task decoding analysis in the SM1 hand ROI, SMA, PMd, and PMv using the scikit-learn python library (Pedregosa et al., 2011) and nilearn (Abraham et al., 2014). We trained a classification algorithm to decode what finger was moved in each trial using the motor execution data. We then used this trained classifier to decode the motor imagery trials, i.e., which finger participants mentally activated. To create the training and test data, we computed single-trial parameter estimates using an HRF-based first-level GLM in SPM12 (http://www.fil.ion.ucl.ac.uk/spm/) using SPM's default parameters. The design matrix consisted of individual regressors for each motor imagery and motor execution trial. This resulted in 48 parameter estimates per finger, session, and participant for motor imagery and 30 for motor execution. Note that for motor execution, only thumb, index, and little finger trials were included. Ring and middle finger trials were modeled as regressors of no interest, as they were not analyzed further for the present study. We added the same nuisance regressors as described in the univariate analysis section. Next, we extracted the voxel-wise parameter estimates below the specified SM1 hand ROI, SMA, PMd, and PMv, separately for each of these ROIs, trial, and participant. To ensure that a classifier can reliably decode executed finger movements, we first conducted a leave-one-run-out cross-validation within the motor execution condition using all runs of the pre- and post-training fMRI sessions, separately for each participant. For that, we scaled the data of the training data in a fold (i.e., 11 out of 12 runs) runs with the StandardScaler from the scikit-learn python library and trained a Support Vector Machine (SVM) with a linear kernel and default parameters of *C* = 1 and l2 regularization. We then applied the StandardScaler fitted on the eleven training runs on the left-out run and predicted the trials of this left-out run. We repeated this until each run once served as the left-out run. The classifier performance was based on the average classification accuracy from the cross-validation (Fig. 7). To define the chance level, we generated a null distribution based on 1,000 random permutations of the trial labels (i.e., “thumb,” “index,” “little”) for each participant. Then we computed an empirical *p* value to evaluate the probability that the classification accuracy score was obtained by chance. For that, we divided the number of permutation-based classification accuracies that were greater than or equal to the true score +1, by the number of permutations + 1. To determine statistical significance at group level, we combined the empirical *p* values of each participant for each ROI separately using Fisher's method (Fisher, 1992).

For the cross-task classification, we scaled the beta estimates across all runs of both the pre- and post-training sessions for each participant but separately for the motor execution and imagery trials. Next, we trained an SVM with linear kernel and default parameters on all motor execution trials and tested it on all motor imagery trials, separately for the two sessions, to compare pre- to post-training decoding accuracy. To determine the empirical chance level, we shuffled the labels of the test set (i.e., motor imagery trials). We corrected the *p* values for multiple comparisons within each group and ROI using the false discovery rate (FDR).

### Statistical analyses

Statistical analyses were performed in R v.4.3.1 (R Core Team) and JASP v. 0.18.3 (JASP Team 2024). We used R packages lme4 (Bates et al., 2015) and lmertest (Kuznetsova et al., 2017) to compute linear mixed-effects models. We defined group (NF, control), session (pre-training, post-training), or motor imagery condition (target, nontarget) as fixed effects and participant as a random effect. For each linear mixed-effects model, we evaluated the expected against observed residuals for normality and homoscedasticity using the R package DHARMA (Hartig, 2022) and did not find any violations. If the model revealed a significant interaction of the fixed effects, we computed post hoc contrasts with the R package emmeans (Lenth, 2024). As we computed only one post hoc contrast for each data set (i.e., each group), no correction for multiple comparisons was applied. For all other tests, we checked the data for violations against normality using the Shapiro–Wilk test. We then used standard classical parametric or nonparametric tests accordingly. We used two-tailed tests for all reported statistical analyses. The significance level alpha was set to 0.05 for all inferential tests. We further used Bayesian tests (with default settings in JASP) to provide evidence for or against the null hypothesis and reported the Bayes factor BF_10_ following conventional cutoffs (Dienes, 2014).

Outliers were defined as >2.5 SD from the group average. For the MEP target ratio, we tested for outliers only in the pre-training session and classified one participant of the NF group as an outlier. Removing this participant did not impact the conclusions of our statistical analysis (Fig. 3*A*).

We used the R package effectsize (Ben-Shachar et al., 2020), to compute Cohen's *d* based on *F* and *t* values from linear mixed-effects models and emmeans for effect sizes of post hoc contrasts, or we computed the effect sizes in JASP. Note that for negative *t* values, we report effect sizes based on the absolute value. For Mann–Whitney tests, we report the rank biserial (*r*_b_) instead of Cohen's *d* as effect size.

## Results

We investigated the neural underpinnings of learning through motor imagery-based NF training. Specifically, we used TMS-NF training to enhance mental “finger individuation,” i.e., the selective facilitation of single finger muscles without producing overt movements (as in Mihelj et al., 2021): We instructed 16 participants to kinaesthetically imagine selective movements of the right thumb, index, or little finger. During motor imagery, we applied a TMS pulse over the contralateral M1 and computed the peak-to-peak amplitude of the TMS-evoked MEPs in the three right hand finger muscles. We then provided visual feedback representing MEP amplitudes normalized to rest (Fig. 1*A*). We trained participants in four TMS-NF sessions taking place on separate days. We gradually increased task difficulty over the training sessions by transitioning from a blocked to an interleaved trial order. All participants were able to successfully modulate corticospinal excitability for individual finger muscles in these training sessions (Fig. 2). We measured motor imagery performance pre and post TMS-NF training to quantify improvements in mental finger individuation. We further assessed plasticity of intracortical circuits in M1 induced by TMS-NF training using paired-pulse TMS protocols pre- and post-training. Finally, we assessed plasticity of neural finger representations in sensorimotor areas using fMRI pre- and post-training. A control group (*n* = 16) participated in identical pre and post measures as the NF group but did not undergo any TMS-NF training (Fig. 1*B*).

10.1523/JNEUROSCI.2189-24.2025.f1-1 Figure 1-1 Verbatim instructions for motor imagery tasks. These instructions were provided at the beginning of the first session (TMS pre-training session) where participants did not receive any feedback yet and were identical for the NF group and control group. Download Figure 1-1, DOCX file.

10.1523/JNEUROSCI.2189-24.2025.f1-2 Figure 1-2 Self-reported strategies used in TMS-NF training. Different strategies that were reported by participants in the NF group (*n* = 16) and number of participants that have used these strategies, separately for each finger. Please note that participants were allowed to use multiple strategies. All reported strategies involved motor imagery. The strategies in the upper block involve additional imagined sensory feedback by touch or pressure, while the strategies in the bottom block include rather imagined proprioceptive or thermoceptive feedback. Download Figure 1-2, DOCX file.

10.1523/JNEUROSCI.2189-24.2025.f1-3 Figure 1-3 Self-reported strategies in the fMRI pre- and post-training sessions. Strategies are listed separately for each participant, sorted for the NF and control group. Please note that we only started to collect self-report of strategies in the fMRI session after study onset (for NF group from participant nr. 9, and for control group from participant nr. 4 on). For participants in the NF group, the strategies from the post-training session corresponds to the strategies used in the TMS-NF training. Download Figure 1-3, DOCX file.

### TMS-NF training improves mental finger individuation

We first tested whether motor imagery performance changed from pre to post TMS-NF training using a task identical to that used during TMS-NF training, but with occluded feedback. We quantified motor imagery performance as the MEP target ratio, i.e., the ratio between the normalized MEP of the cued target finger muscle and the larger normalized MEP of the two nontarget finger muscles (Mihelj et al., 2021). As such, an MEP target ratio greater than 1 indicates a finger-selective upregulation of corticospinal excitability. We found that the NF group improved motor imagery performance from pre- to post-training (*t*_(30.1)_ = −2.55, *p* = 0.02, Cohen's *d* = 0.93, 95% CI for Cohen's *d*: [0.18, 1.69]), whereas the control group did not (*t*_(29.6)_ = 0.57, *p* = 0.58, Cohen's *d* = 0.20, 95% CI for Cohen's *d*: [−0.52, 0.93]; significant session (pre-training, post-training) by group (NF, control) interaction: *F*_(1,30.9)_ = 4.69, *p* = 0.04, Cohen's *d* = 0.78, 95% CI for Cohen's *d*: [0.04, 1.50]; Fig. 3*A*). There was no significant difference in motor imagery performance between the groups in the pre-training session (*U* = 152, *p* = 0.38, *r*_b_ = 0.19, 95% CI for *r*_b_: [−0.21, 0.53]; BF_10_ = 0.42 indicating anecdotal evidence for no difference between the NF and the control groups). The bgEMG target ratio, which was added as a covariate, did not significantly contribute to the prediction of motor imagery performance (*F*_(1,48.3)_ = 0.51, *p* = 0.48, Cohen's *d* = 0.21, 95% CI for Cohen's *d*: [−0.36, 0.77]). We detected one outlier participant in the NF group based on the pre-training session. Removing this participant did not change interpretation of our results. Additionally, we repeated the analysis by computing a ratio based on the data visualized in Figure 3*C*, i.e., the ratio between the normalized MEP of the target finger and the averaged normalized MEP of the two nontarget fingers. By including all three MEPs in the ratio, we obtained similar results (Fig. 3*B*). These findings confirm that training with TMS-NF improved mental finger individuation that translated to later sessions where participants did not receive any NF.

### Intracortical inhibitory circuits are tuned following TMS-NF training

**Figure 4.**
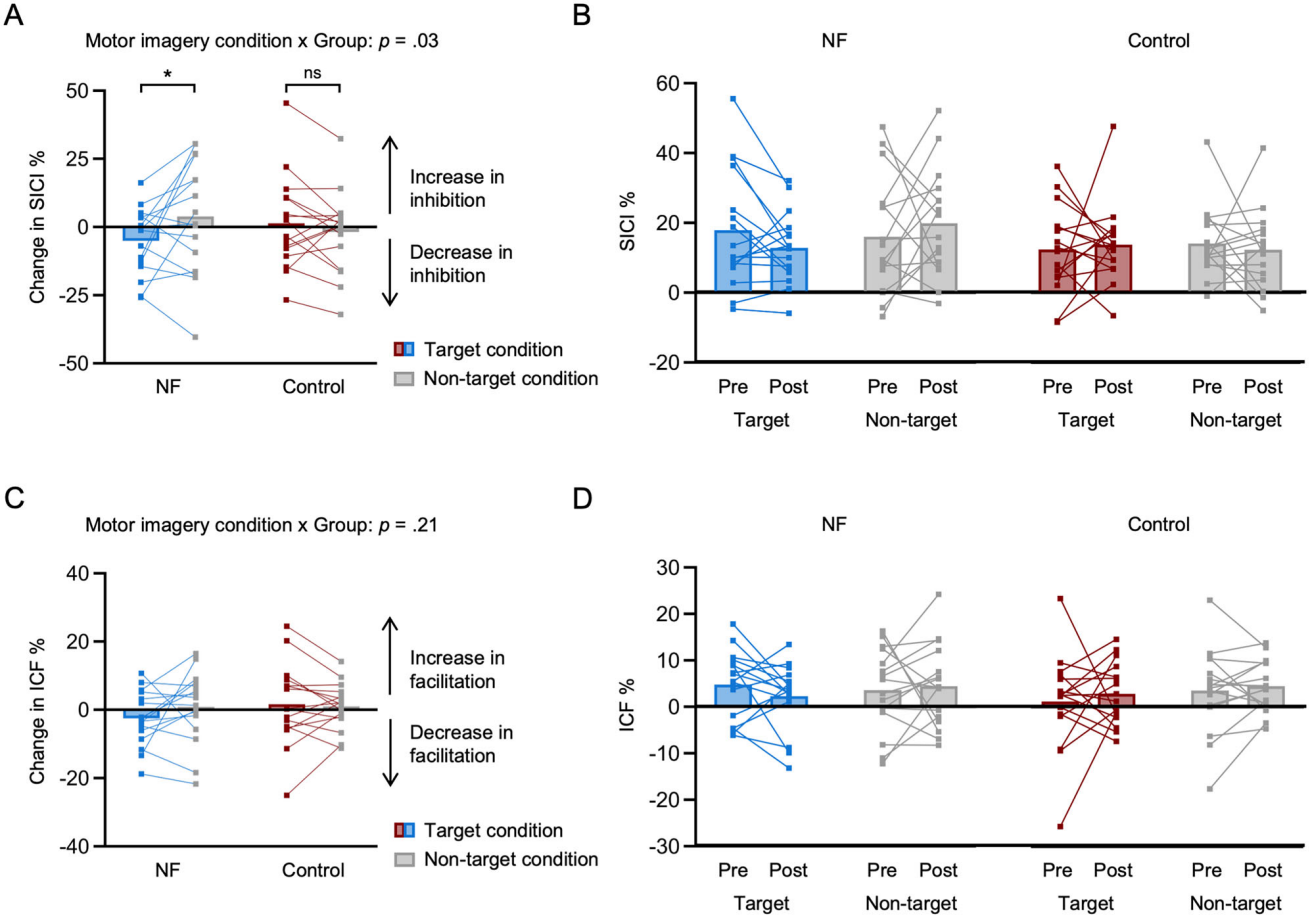
Intracortical inhibitory circuits are tuned after TMS-NF training. ***A***, Changes in short-interval intracortical inhibition (SICI) after TMS-NF training for the NF (blue) and control (red) groups. SICI was assessed with adaptive threshold hunting to determine the minimum testing stimulus intensity needed to elicit an MEP with an amplitude of at least 50% of the maximum MEP in 50% of trials. If the threshold did not converge reliably, the protocol was repeated (see Extended Data Fig. 4-1 for number of repetitions per participant). We measured SICI in the right index finger muscle during two motor imagery conditions: index as the target finger (motor imagery of index finger movements) versus index as an adjacent nontarget finger (motor imagery of thumb movements). SICI is expressed as the % increase in the required testing stimulus intensity in the SICI protocol compared with a nonconditioned single pulse protocol during the same motor imagery condition. Positive scores indicate an increase in inhibition and negative scores indicate a decrease in inhibition after TMS-NF training. ***B***, SICI for the pre- and post-training sessions separately. This data is shown for visualization purposes only. ***C***, No changes in intracortical facilitation (ICF) from pre- to post-training. ICF is expressed as the % increase in the required testing stimulus intensity in the ICF protocol compared with a nonconditioned single pulse protocol during the same motor imagery condition. Positive scores indicate an increase in facilitation and negative scores indicate a decrease in facilitation after TMS-NF training. We did not observe any changes in facilitation from pre- to post-training for the target relative to the nontarget condition in any group: no group by motor imagery condition interaction: *F*_(1,30)_ = 1.64, *p* = 0.21, Cohen's *d* = 0.47, 95% CI for Cohen's *d*: [−0.26, 1.19]. ***D***, ICF for the pre- and post-training sessions separately. This data is shown for visualization purposes only. Squares depict data of individual participants. *p < 0.05; ns, nonsignificant.

To investigate neuroplasticity induced by TMS-NF training, we first tested for changes in neurophysiological circuits. As intracortical inhibition and facilitation are highly relevant in shaping motor representations for skilled finger individuation, we aimed to investigate if there was a release of SICI (and/or increase of ICF) from pre- to post-training for a mentally activated versus nonactivated finger. We measured MEPs in the right index finger muscle (FDI) and applied the SICI and ICF paired-pulse TMS protocols while participants imagined moving either their index finger or their thumb. This resulted in two motor imagery conditions where the index finger was either the target or a nontarget finger. We calculated the pre- to post-training change in SICI such that positive scores indicate an increase, and negative scores indicate a decrease in inhibition after TMS-NF training. We found that the change of SICI after training significantly differed for the target compared with the nontarget condition in the NF group (*t*_(30)_ = −2.39, *p* = 0.02, Cohen's *d* = 0.85, 95% CI for Cohen's *d*: [0.13, 1.56]). In other words, we observed a decrease in intracortical inhibition when the index finger was the target (i.e., mentally activated) finger, compared with when it was the nontarget finger. No such effect was found in the control group (*t*_(30)_ = 0.86, *p* = 0.39, Cohen's *d* = 0.31, 95% CI for Cohen's *d*: [−0.41, 1.02]; significant motor imagery condition by group interaction: *F*_(1,30)_ = 5.29, *p* = 0.03, Cohen's *d* = 0.84, 95% CI for Cohen's *d*: [0.09, 1.58]; Fig. 4*A*,*B*). This finding suggests that tuning of intracortical inhibition contributes to improved mental finger individuation. Analogous analyses were performed with ICF, but we did not find any significant effects of TMS-NF training (Fig. 4*C*,*D*).

10.1523/JNEUROSCI.2189-24.2025.f4-1 Figure 4-1 Number of repetitions for the paired-pulse TMS protocols in the pre- and post-training TMS sessions. Download Figure 4-1, DOCX file.

### Individual finger motor imagery activates a frontoparietal network

**Figure 5.**
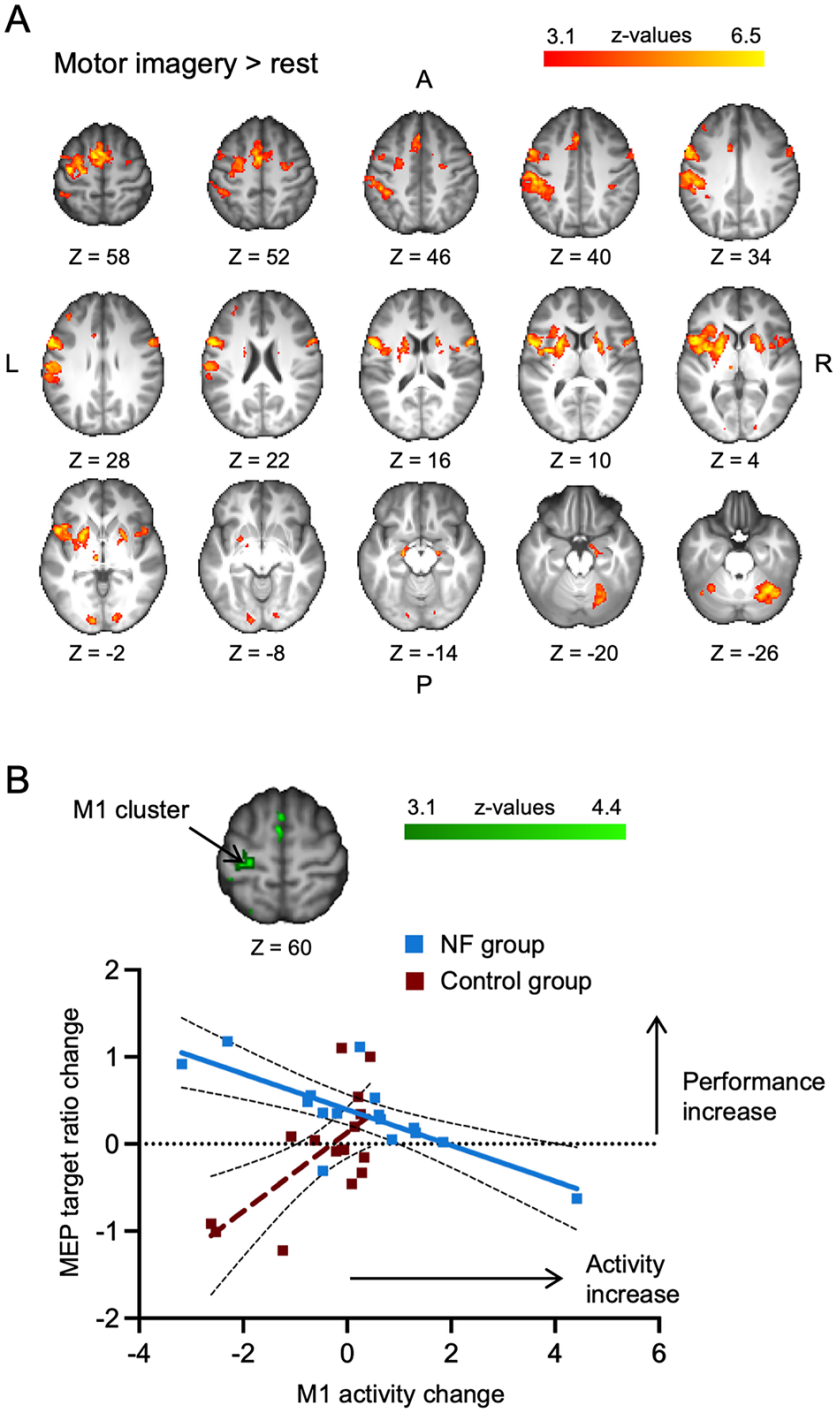
M1 activity changes predict motor imagery performance changes. ***A***, Whole-brain maps showing the overall activity during motor imagery (i.e., across all fingers and both groups) in the pre-training session. Individual finger motor imagery activated a frontoparietal network and subcortical structures that are typical for motor imagery (see Extended Data Fig. 5-1 for a list of all activated clusters and Extended Data Fig. 5-2 for pre- to post-training changes). ***B***, The relationship between pre- to post-training changes in M1 activity and motor imagery performance differs between the NF (blue) and control (red) groups (see Extended Data Fig. 5-3 for a list of all significant clusters). Changes in activity level in the M1 cluster (*z* values) are depicted on the *x*-axis, with positive values showing an increase in activity from pre- to post-training. Changes in motor imagery performance (i.e., MEP target ratio) are depicted on the *y*-axis, with positive values indicating an improvement from pre- to post-training. Squares depict data of individual participants, colored lines show the best fit, and white dotted lines show the 95% confidence bands.

We then investigated which areas were active during individual finger motor imagery by analyzing univariate brain activity during motor imagery versus rest in the pre-training fMRI session. Our results confirmed that individual finger motor imagery (across all fingers and groups) activated a frontoparietal network that is typically observed during motor imagery (Hardwick et al., 2018; Hétu et al., 2013; Fig. 5*A*). We observed activity in contralateral PMd and PMv with activity stretching into the M1 hand area, the inferior and superior parietal lobules, and bilateral SMA (see Extended Data Fig. 5-1 for a full list of activated clusters). We then computed pre- to post-training changes in univariate activity levels during motor imagery. A whole-brain analysis did not reveal any significant differences from pre- to post-training between groups (Extended Data Fig. 5-2). Next, we aimed to assess the univariate activity correlates of improved mental finger individuation. To do so, we investigated whether the prediction of pre-to-post changes in motor imagery performance through pre-to-post changes in activity differed between the two groups. We found a significant cluster in M1 that overlapped with our main SM1 hand area ROI (see Extended Data Fig. 5-3 for all significant clusters). We then extracted the pre-to-post activity changes under this M1 cluster for each participant and correlated it with the corresponding MEP target ratio change (Fig. 5*B*). For the NF group, we found a negative correlation, such that an increase in motor imagery performance was associated with a decrease in M1 activity (*r*_Pearson_ = −0.75, *p* < 0.001, 95% CI: [−0.91, −0.40]). For the control group, we observed a trend toward a positive correlation (*r*_Spearman_ = 0.48, *p* = 0.06, 95% CI: [−0.02, 0.79]).

10.1523/JNEUROSCI.2189-24.2025.f5-1 Figure 5-1 Motor imagery network. Activation clusters, corresponding size, anatomical region, FW-corrected p-value for multiple comparisons, peak coordinate in MNI space, and maximum z-value of the reported contrast of motor imagery (pre-training session, across all fingers and groups) vs rest thresholded at Z > 3.1. Reported anatomical labels were determined using the Jülich Histological (Eickhoff et al., 2005), the Harvard-Oxford cortical (Desikan et al., 2006) and subcortical structural (Frazier et al., 2005), and the probabilistic cerebellar atlases (Diedrichsen et al., 2009), correspond to the location of maxima within each cluster. Download Figure 5-1, DOCX file.

10.1523/JNEUROSCI.2189-24.2025.f5-2 Figure 5-2 Pre- to post-training changes. Activation clusters, corresponding size, anatomical region, FW-corrected p-value for multiple comparisons, peak coordinate in MNI space, and maximum z-value of the reported pre- to post-training contrasts thresholded at Z > 3.1. Reported anatomical labels were determined using the Jülich Histological (Eickhoff et al., 2005), the Harvard-Oxford cortical (Desikan et al., 2006) and subcortical structural (Frazier et al., 2005), and the probabilistic cerebellar atlases (Diedrichsen et al., 2009), correspond to the location of maxima within each cluster. Download Figure 5-2, DOCX file.

10.1523/JNEUROSCI.2189-24.2025.f5-3 Figure 5-3 Interaction analysis. Activation clusters, corresponding size, anatomical region, FW-corrected p-value for multiple comparisons, peak coordinate in MNI space, and maximum z-value of the reported interaction analysis where the relationship between pre- to post-training changes and motor imagery performance changes differs between the NF and control groups, thresholded at Z > 3.1. Reported anatomical labels were determined using the Jülich Histological (Eickhoff et al., 2005), the Harvard-Oxford cortical (Desikan et al., 2006) and subcortical structural (Frazier et al., 2005), and the probabilistic cerebellar atlases (Diedrichsen et al., 2009), correspond to the location of maxima within each cluster. Download Figure 5-3, DOCX file.

### Neural finger representations activated by motor imagery become more distinct following TMS-NF training

**Figure 6.**
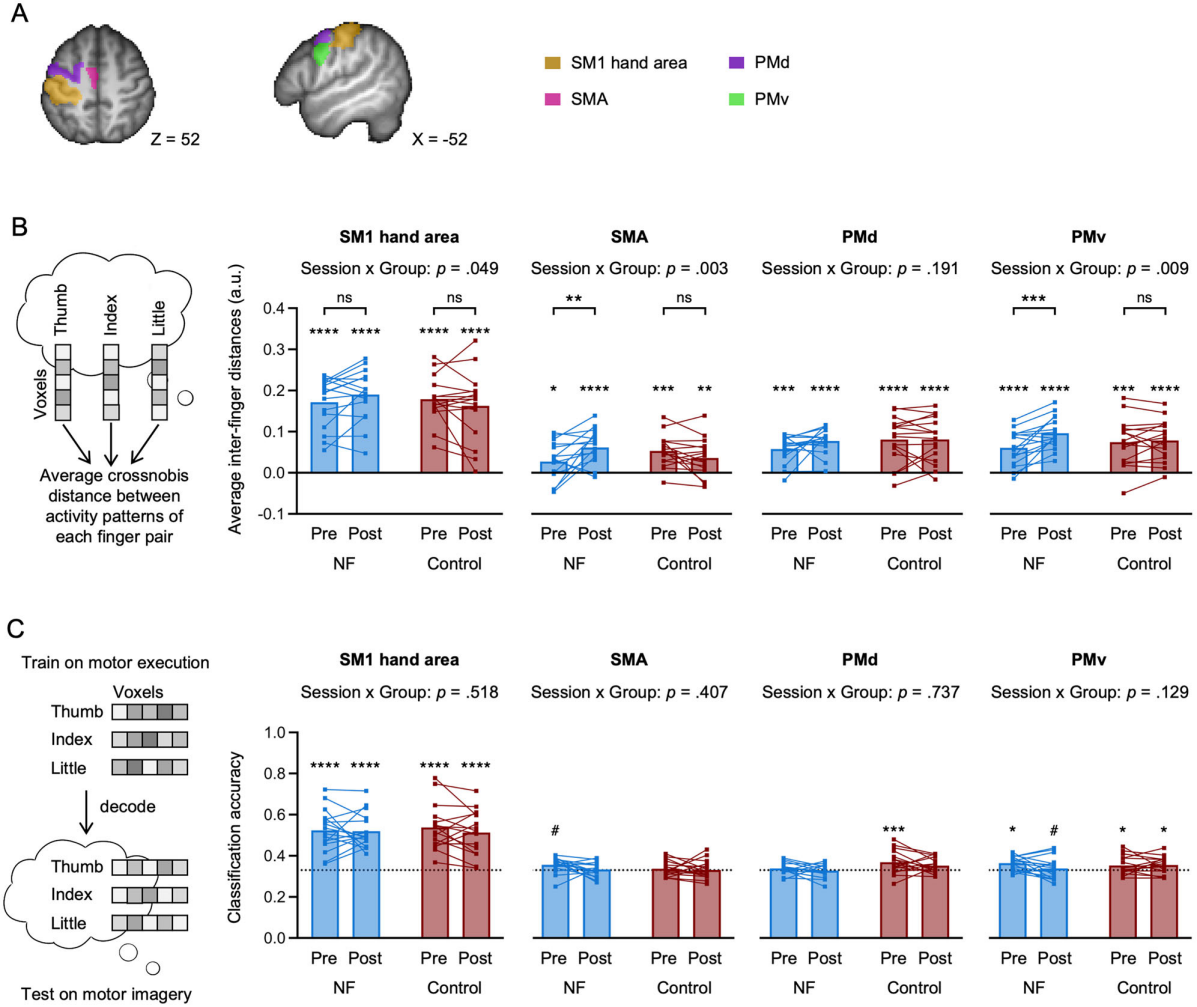
Finger representations activated by individual finger motor imagery become more distinct following TMS-NF training but do not become more similar to motor execution. ***A***, Anatomically defined regions of interest (ROIs) used for multivariate pattern analysis. ***B***, Average inter-finger distances for individual finger motor imagery in the SM1 hand, SMA, PMd, and PMv ROIs for the NF (blue) and control (red) groups. The distance is computed as the average cross-validated Mahalanobis (crossnobis) distance between activity patterns elicited by individual finger motor imagery across finger pairs. Asterisks directly above the bars indicate significant differences from 0 (FDR-corrected within each ROI and group). ***A***, Cross-task classification accuracy. A linear support vector machine was trained separately for each participant on all motor execution trials across both the pre- and post-training sessions to predict the motor imagery trials in the pre- and post-training sessions separately. The dotted line represents the empirical chance level (33.33%). Asterisks refer to the statistical difference of classification accuracy from the empirical chance level (FDR-corrected within each ROI and group). Squares depict data of individual participants. ****p < 0.0001; ***p < 0.001; **p < 0.01; *p < 0.05; ^#^*p* < 0.10; ns, nonsignificant.

Next, we performed an in-depth investigation of plasticity of fine-grained finger representations in SM1 and exploratively in secondary motor areas (Fig. 6*A*) with MVPA. To examine the multivariate distances between activity patterns elicited by individual finger motor imagery in an anatomically defined ROI, we used RSA. This is particularly advantageous in the case of overlapping (finger) representations as in SM1 (Ejaz et al., 2015; Ogawa et al., 2019; Gooijers et al., 2022). We expected that after TMS-NF training, the information content distinguishing between individual finger motor imagery would increase in SM1, resulting in increased average inter-finger distances.

We observed that average inter-finger distances were greater than 0 in all ROIs for all sessions and groups (all *p*_(FDR)_ < 0.033), indicating that the activity patterns in SM1 and all tested secondary motor areas contained finger-specific information. We found that finger representations activated by motor imagery became more distinct in SM1 following TMS-NF training for the NF group compared with the control group (significant session by group interaction; *F*_(1,30)_ = 4.22, *p* = 0.049, Cohen's *d* = 0.75, 95% CI for Cohen's *d*: [0.00, 1.48]; Fig. 6*B*). However, post hoc contrasts comparing the pre- to post-training sessions separately for the groups did not reach significance (NF group: *t*_(30)_ = −1.56, *p* = 0.13, Cohen's *d* = 0.55, 95% CI for Cohen's *d*: [−0.17, 1.28]; control group: *t*_(30)_ = 1.34, *p* = 0.19, Cohen's *d* = 0.47, 95% CI for Cohen's *d*: [−0.25, 1.20]). In secondary motor areas, we found significant session by group interactions for SMA (*F*_(1,30)_ = 10.56, *p* = 0.003, Cohen's *d* = 1.19, 95% CI for Cohen's *d*: [0.40, 1.95]), and PMv (*F*_(1,30)_ = 7.74, *p* = 0.009, Cohen's *d* = 1.02, 95% CI for Cohen's *d*: [0.25, 1.77]), but not for PMd (*F*_(1,30)_ = 1.79, *p* = 0.19, Cohen's *d* = 0.49, 95% CI for Cohen's *d*: [−0.24, 1.21]). Average inter-finger distances in SMA (*t*_(30)_ = −3.07, *p* = 0.005, Cohen's *d* = 1.09, 95% CI for Cohen's *d*: [0.35, 1.82]) and PMv (*t*_(30)_ = −4.48, *p* = 0.0001, Cohen's *d* = 1.58, 95% CI for Cohen's *d*: [0.81, 2.36]) increased significantly from pre- to post-training for the NF group but not for the control group (SMA: *t*_(30)_ = 1.53, *p* = 0.14, Cohen's *d* = 0.54, 95% CI for Cohen's *d*: [−0.18, 1.26]; PMv: *t*_(30)_ = −0.54, *p* = 0.59, Cohen's *d* = 0.19, 95% CI for Cohen's *d*: [−0.53, 0.91]). Any observed interactions were not driven by pre-training group differences (SM1 hand area: *U* = 124, *p* = 0.90, *r*_b_ = −0.03, 95% CI for *r*_b_: [−0.41, 0.36]; BF_10_ = 0.36 indicating anecdotal evidence for no difference between the NF and the control groups; SMA: *t*_(30)_ = 1.71, *p* = 0.10, Cohen's *d* = 0.60, 95% CI for Cohen's *d*: [−0.11, 1.31], BF_10_ = 1.01 indicating no evidence for either hypothesis; PMv: *t*_(30)_ = 0.77, *p* = 0.45, Cohen's *d* = 0.27, 95% CI for Cohen's *d*: [−0.43, 0.97], BF_10_ = 0.42 indicating anecdotal evidence for no difference between groups).

### Activity patterns elicited during individual finger motor imagery do not become more similar to those observed during motor execution after TMS-NF training

**Figure 7.**
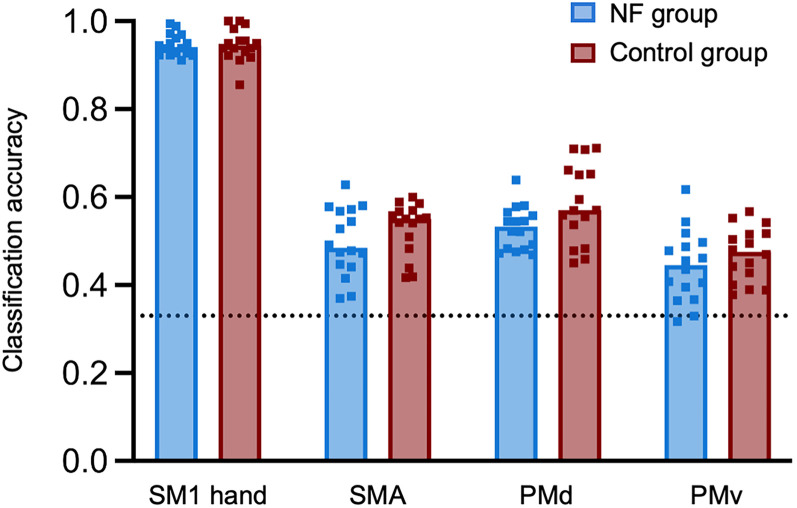
Average classification accuracy from leave-one-run-out cross-validation within motor execution trials across the pre- and post-training sessions, separately for all ROIs and both groups (NF group, blue; control group, red). The dotted line represents the empirical chance level (33.33%). Classification accuracy for each ROI and group was significantly different from the empirical chance level (all *p* < 0.0001). Squares depict data of individual participants.

To investigate whether neural activity patterns elicited by individual finger motor imagery became more similar to those observed during motor execution following TMS-NF training, we performed a cross-task decoding analysis. Specifically, we trained a linear support vector machine to decode fingers during the motor execution task (i.e., paced individual finger tapping; Fig. 7) and tested whether this decoder could be generalized to the motor imagery task. If there is shared information in the activity patterns elicited by imagined and executed finger movements in a given ROI, then this would be reflected in a cross-task classification accuracy above chance level. Furthermore, if the activity patterns elicited by motor imagery would become more similar to motor execution after TMS-NF training, then the cross-task classification would increase from pre- to post-training.

We found consistent classification accuracies greater than chance for all sessions and groups for the SM1 hand area but not for secondary motor areas (Fig. 6*C*). However, the cross-task classification accuracy in the SM1 hand area did not differ significantly across sessions or groups (all *p* > 0.36 for main effects and group by session interaction). Bayesian tests provided moderate evidence for the NF group (BF_10_ = 0.26) and anecdotal evidence for the control group (BF_10_ = 0.50) for no change from pre- to post-training sessions.

### Neural changes do not directly predict changes in motor imagery performance

Finally, we explored whether the improved motor imagery performance in the NF group (i.e., pre- to post-training change in MEP target ratio) related to our main neural outcome measures (i.e., SICI % changes in the target condition, changes of inter-finger distances in the SM1 hand area, and changes in cross-task classification accuracy changes in the SM1 hand area). A multiple linear regression revealed that the changes in the measured neural mechanisms paralleled an improvement in motor imagery performance in the NF group but did not directly predict the observed performance changes (multiple *r*^2^ = 0.12; average inter-finger distances: *t* = 1.13, *p* = 0.28; cross-task classification: *t* = −0.90, *p* = 0.39; SICI %: *t* = 0.18, *p* = 0.86). Similar results were found when including both groups in the analysis (i.e., NF and control groups; multiple *r*^2^ = 0.05; average inter-finger distances: *t* = 0.43, *p* = 0.67; cross-task classification: *t* = 0.52, *p* = 0.61; SICI %: *t* = 0.71, *p* = 0.49).

## Discussion

In this study, we investigated neuroplastic changes induced by mental finger individuation training that was guided by TMS-NF. We found that TMS-NF training enabled participants to selectively upregulate the corticospinal excitability of a target finger while simultaneously downregulating the corticospinal excitability of other finger representations, even in the absence of feedback. Improved mental finger individuation was accompanied by tuning of intracortical inhibitory circuits: After TMS-NF training, GABA_A_-ergic inhibition of a finger representations was reduced when that finger was mentally activated, relative to when a different finger was mentally activated. We further found that through TMS-NF training, activity patterns underpinning individual finger motor imagery became more distinct in SM1, SMA, and PMv compared with the control group. With these results, we substantially extend our previous work (Mihelj et al., 2021), where we combined our TMS-NF approach with electroencephalography (EEG) to investigate online effects of TMS-NF on sensorimotor brain rhythms. In the present study, we demonstrate effects that persist beyond the TMS-NF training period, influencing both motor imagery performance and, importantly, measures of neuroplasticity.

At the neurophysiological level, we found changes in intracortical inhibition after TMS-NF training. In the NF group, there was a release of intracortical inhibition measured in the mentally activated target finger muscle, compared with an increase in intracortical inhibition during motor imagery of another (nontarget) finger. Intracortical inhibition, assessed with SICI, is thought to be driven by inhibitory interneurons in M1 (Peurala et al., 2008) and is crucial for the fine-tuned activation and suppression of motor representations (Liepert et al., 1998; Stinear and Byblow, 2003b). Importantly, previous work has suggested that a release of SICI during BCI-NF training modulates the activation of adjacent sensorimotor representations rather than simply inducing a general increase of corticospinal excitability (Takemi et al., 2018), similarly as during executed movements (Stinear and Byblow, 2003b; Takemi et al., 2018). The modulatory effects on SICI during mental finger individuation observed in our study suggest that the tuning of intracortical inhibitory mechanisms may have “shaped” motor imagery finger representations during TMS-NF training.

The finding that the TMS-NF group learned to activate individual finger representations more selectively through training further aligns with our fMRI results. Specifically, following TMS-NF training, activity patterns elicited by motor imagery in SM1 became more finger-specific (i.e., inter-finger distances increased from pre- to post-training) compared with the control group that did not undergo any TMS-NF training. However, in both the NF and the control groups, pre-to-post changes in SM1 representations were subtle and did not reach significance. In contrast, we observed a clear pattern of increased inter-finger distances from pre- to post-training in SMA and PMv for the NF group.

The subtle changes we observed in SM1 are consistent with previous research on the plasticity of finger representations, suggesting that SM1 representations are generally stable or only slightly malleable. For instance, a study in which participants trained specific finger movement sequences did not find altered finger representations in S1/M1 (Beukema et al., 2019). However, finger movement representations in M1 and/or S1 have been shown to be slightly altered after drastic (temporary) changes in sensorimotor experiences (Kolasinski et al., 2016b), such as lifelong practice of playing instruments in professional musicians (Ogawa et al., 2019), or temporary finger deafferentation (Wesselink et al., 2022). Notably, while finger representations are preserved after long-term sensorimotor deprivation, such as following an arm amputation or chronic SCI, the “typicality” of these finger representations has been correlated with clinical characteristics, such as time since injury (SCI; Kikkert et al., 2021) or the number of fingers with kinaesthetic phantom sensations (amputees; Wesselink et al., 2019). In contrast, online BCI-NF has been shown to alter finger representations within a single session (Oblak et al., 2021). Our findings support the notion that BCI-NF training, through gaining volitional control over brain activity, can have effects on (motor imagery) finger representations already after a few sessions, which even persist beyond the training period. While most previous studies focused on the neuroplasticity of finger representations in SM1, our results suggest that the effects of BCI-NF on finger representations may be even more pronounced in secondary motor areas.

We further found that individual finger motor imagery and execution activate (partially) the same fine-grained finger representations in SM1: A decoder trained on executed finger movements successfully generalized to imagined finger movements. However, this neural similarity did not change after TMS-NF training. A potential increase in neural similarity might have been masked by task differences. Developing personalized motor imagery strategies was an integral part of the TMS-NF training, and as a result, participants used strategies that varied from, for example, button pressing, making circles with the cued finger, and touching a surface, to finger abduction (see Extended Data Fig. 1-3 for a complete list of strategies). In contrast, movement execution consisted of a paced button press task. These task differences may also explain why cross-task decoding was not successful in secondary motor areas, as activity patterns in these areas have been shown to be distinct for different types of (imagined) actions within the same effector (Park et al., 2015; Zabicki et al., 2017). Furthermore, it has been proposed that motor imagery and execution rely on different neural substrates within M1 (Persichetti et al., 2020). Specifically, motor imagery is thought to be represented in superficial layers, while motor execution may be represented in both superficial and deep layers (Persichetti et al., 2020; Neige et al., 2022a). Thus, the observed pre- to post-training changes in activity patterns elicited through motor imagery might not be directly reflected in higher neural similarity with movement representations.

Improvements in motor imagery performance through TMS-NF training were associated with decreased M1 activity during motor imagery. This might reflect more efficient or more targeted activation of finger representations, in line with previous research showing that accentuated sensory representations are associated with lower activity levels (Yon et al., 2018). Our main outcome measures, i.e., pre-to-post changes in intracortical inhibition, in motor imagery finger representations in SM1, and in neural similarity of motor imagery and motor execution finger representations, were not directly associated with better motor imagery performance. While not directly associated statistically, the change in motor imagery performance after TMS-NF training may still have been driven by the observed neuroplasticity. Motor imagery performance was relatively variable, especially in the absence of visual feedback. This variability was present in the pre-training assessment of motor imagery performance but may also have extended to the fMRI and paired-pulse TMS measures in the pre- as well as the post-training sessions: While participants improved motor imagery performance after the relatively short training period, more training sessions may have led to more stability in mental finger individuation. Thus, variability in the pre-to-post differences may explain why the neuroplasticity and performance measures were not directly associated. Furthermore, neuroplasticity as reflected in changes in intracortical inhibition and motor imagery finger representations may follow a different time scale than performance changes. Motor imagery performance has been shown to improve swiftly upon TMS-NF training (Majid et al., 2015; Koganemaru et al., 2018; Ruddy et al., 2018; Matsuda et al., 2021; Mihelj et al., 2021), which may precede the neuroplastic changes. Finally, it is possible that the neuroplastic changes may be related to the duration of training itself rather than task improvements, as has been previously suggested in a training study (Scholz et al., 2009).

We suggest that the observed neuroplastic changes in intracortical inhibition and finger representations induced by TMS-NF training were driven by an interplay of processes (Simon et al., 2021). First, use-dependent plasticity in SM1 has been frequently demonstrated in TMS studies for motor execution (Classen et al., 1998; Mawase et al., 2017) and motor imagery tasks (Ruffino et al., 2019; Yoxon and Welsh, 2019; Neige et al., 2022b). It is possible that such use-dependent plasticity, possibly driven by long-term potentiation (LTP)-like mechanisms, has been triggered by repeated practice with TMS-NF (Ruffino et al., 2017). Second, gaining control of BCI-NF via motor imagery may reflect skill learning that involves a neural network beyond SM1. Neuroplasticity in SM1 may emerge due to inter- and intrahemispheric connections with various other, higher-order, brain areas, such as premotor and parietal association areas that are consistently activated during motor imagery (Hétu et al., 2013; Ruddy et al., 2017; Hardwick et al., 2018). Our finding of increased average inter-finger distances during motor imagery in SMA and PMv following TMS-NF training supports this notion. Previous work indicates that controlling BCI-NF via motor imagery is a skill that, once acquired, can be maintained over extended periods without training (Ruddy et al., 2018), further suggesting that skill learning may be involved in BCI-NF learning. Finally, research has shown that individuals can activate S1 finger representations by merely directing attention to individual fingers (Puckett et al., 2017 ). Participants may have targeted motor imagery representations more selectively through improving attentional processes. Importantly, these potential mechanisms are not mutually exclusive, and it is likely that use-dependent, skill learning-dependent, and attentional processes jointly contributed to the observed changes following TMS-NF training.

The neural changes induced by TMS-NF training demonstrate its promise to restore fine motor control. Regaining hand functions has been reported as one of the most important therapy goals by tetraplegic and stroke patients (Anderson, 2004; Collinger et al., 2013; Hay et al., 2022). TMS-NF might offer a rehabilitation strategy that can be employed already in the early stages after a stroke or SCI, even in the absence of overt movements (Liang et al., 2020). Importantly, our findings of neuroplasticity open new avenues to investigate the extension of TMS-NF to shape sensorimotor representations. TMS-NF training could be implemented, potentially already pre-BCI use, to improve control in BCIs that rely on clearly distinct neural activity patterns, such as EEG-based BCIs.

In summary, our results indicate that the neural underpinnings of finger individuation, a well-known model system for neuroplasticity, can be modified using motor imagery training that is guided by TMS-NF. With this proof-of-principle study, we demonstrate that TMS-NF training can indeed promote neuroplasticity that may be relevant for motor recovery.

## Data Availability

Processed EMG and fMRI data are openly available on the ETH Library Research Collection (https://doi.org/10.3929/ethz-b-000748847).
