# Supplementary material for: TMS-Based Neurofeedback Training of Mental Finger Individuation Induces Neuroplastic Changes in the Sensorimotor System
Source: J Neurosci. 2025 Jul 24;45(35):e2189242025. doi: 10.1523/JNEUROSCI.2189-24.2025 (PMC12392065; doi:10.1523/JNEUROSCI.2189-24.2025)
Supplement: Figure 1-1 — Verbatim instructions for motor imagery tasks. These instructions were provided at the beginning of the first session (TMS pre-training session) where participants did not receive any feedback yet and were identical for the NF group and control group. Download Figure 1-1, DOCX file. [file jneuro-45-e2189242025-s001.docx]

| - Increase activity by vividly imagining to move (feeling not seeing!) the instructed finger of the right hand, for example by imagining:   - to move the finger up/down, left/right.   - to press a button, playing piano, typing on a keyboard.   - pulses in the muscle.   - … - Complex or forceful movements will give more activation than weak or simple movements. - The imagined movement should only involve the instructed finger and no others. |
| --- |
| - Decrease activity for the not instructed fingers, for example by imagining:   - that the other fingers were very cold, as if in a bucket of ice water.   - would not belong to the body or not exist at all.   - … |
| - Imagine as long as you can see the instruction on the screen. There will be one or two TMS pulse(s) during this time. - Ensure no actual muscle activity anywhere in the body. Keep also your face muscles completely relaxed. |
