# Supplementary material for: TMS-Based Neurofeedback Training of Mental Finger Individuation Induces Neuroplastic Changes in the Sensorimotor System
Source: J Neurosci. 2025 Jul 24;45(35):e2189242025. doi: 10.1523/JNEUROSCI.2189-24.2025 (PMC12392065; doi:10.1523/JNEUROSCI.2189-24.2025)
Supplement: Figure 1-2 — Self-reported strategies used in TMS-NF training. Different strategies that were reported by participants in the NF group (n = 16) and number of participants that have used these strategies, separately for each finger. Please note that participants were allowed to use multiple strategies. All reported strategies involved motor imagery. The strategies in the upper block involve additional imagined sensory feedback by touch or pressure, while the strategies in the bottom block include rather imagined proprioceptive or thermoceptive feedback. Download Figure 1-2, DOCX file. [file jneuro-45-e2189242025-s002.docx]

| **Strategy** | **Thumb** | **Index** | **Little** |
| --- | --- | --- | --- |
| imagine to tap or press on button, keyboard/piano key, phone, pillow | 11 | 15 | 10 |
| imagine to move the finger over pillow | 1 | 3 | 1 |
| imagine to pull or plug a string/object (e.g. violin, open a soda can), digging in sand, pinky promise | 2 | 1 | 2 |
| imagine that whole bodyweight is hold by this finger | 0 | 0 | 1 |
| imagine that finger sticked in a (hot or cold) rocky hole, focus on climbing texture | 1 | 1 | 0 |
|  | | | |
| imagine to hold, bend or move the fingers to the left, right, up, downwards, in circles (e.g. thumbs up, point on something, say "no" with index finger, dancing with the finger) | 12 | 11 | 12 |
| imagine that other fingers are not here, only focus on the target finger | 1 | 1 | 1 |
| imagine that finger is heavy/energized and other fingers are light | 1 | 1 | 0 |
| imagine that finger get warm and other fingers get cold OR just the target finger get cold/warm | 2 | 1 | 2 |
| imagine the muscle contraction in the finger | 1 | 0 | 1 |
