# Supplementary material for: TMS-Based Neurofeedback Training of Mental Finger Individuation Induces Neuroplastic Changes in the Sensorimotor System
Source: J Neurosci. 2025 Jul 24;45(35):e2189242025. doi: 10.1523/JNEUROSCI.2189-24.2025 (PMC12392065; doi:10.1523/JNEUROSCI.2189-24.2025)
Supplement: Figure 1-3 — Self-reported strategies in the fMRI pre- and post-training sessions. Strategies are listed separately for each participant, sorted for the NF and control group. Please note that we only started to collect self-report of strategies in the fMRI session after study onset (for NF group from participant nr. 9, and for control group from participant nr. 4 on). For participants in the NF group, the strategies from the post-training session corresponds to the strategies used in the TMS-NF training. Download Figure 1-3, DOCX file. [file jneuro-45-e2189242025-s003.docx]

| **NF group** | **Session** | **Thumb** | **Index** | **Little** |
| --- | --- | --- | --- | --- |
| 9 | pre | dancing with the finger | dancing with the finger | dancing with the finger |
|  | post | feeling the muscle contraction | pressing the index finger against the pillow | dancing with the finger |
| 10 | pre | thumbs up | pointing on something | keyboard |
|  | post | thumbs up  partying of the thumb  pushing something with the finger | pointing on something  partying of the index finger | pushing button  partying of the little finger  moving the finger towards left and right |
| 11 | pre | feeling the thumb close  opening a can of beer with the thumb | flicking a paper ball  pointing at someone  opening a can of beer  rubbing the table | stretching the finger  closing the finger |
|  | post | pushing thumb outwards (in and out) in little movements | pushing index finger outwards | pushing little finger outwards |
| 12 | pre | movements in the joint  movement of the skin due to the movements in all directions | movements in the joint  movement of the skin due to the movements in all directions  -> especially flexion | movements in the joint  movement of the skin due to the movements in all directions  -> especially flexion |
|  | post | pressing space bar, exaggerated extension | pressing key on keyboard | pressing shift on keyboard |
| 13 | pre | thumb stuck and wriggling out  frozen/paralyzed other fingers  moving thumb | finger stuck and wriggling out | sipping a cup of tea like a gentleman |
|  | post | sticking into rocky holes - something hot and cold | move heavily on the climbing, texture feeling | negative incidents involving little finger,  little finger injuries |
| 14 | pre | pushing button inwards | pushing button downwards | pressure downwards |
|  | post | pressure towards left downwards | pressure towards left downwards | pressure downwards |
| 15 | pre | moving up- and downwards | making circles, up- and downward, left and right | scratching on surface |
|  | post | Lighter | pressing | spreading |
| 16 | pre | pushing elevator button | pushing elevator button | pushing elevator button |
|  | post | pressing down | pressing down | pressing down |
|  | | | | |
| **Control group** | **Session** | **Thumb** | **Index** | **Little** |
| 4 | pre | pushing palm | pushing button | *did not recall* |
|  | post | pushing palm | brooding cheese | pushing button |
| 5 | pre | "thumbs up" multiple time | holding up index finger | holding up little finger |
|  | post | typing | typing | typing |
| 6 | pre | moving thumb up- and downwards,  pushing piano key,  typing,  moving thumb over surface | moving index up- and downwards,  pushing piano key,  typing,  moving index over surface | moving little finger up- and downwards,  pushing piano key,  typing,  moving little finger over surface |
|  | post | moving thumb up- and downwards,  pushing piano key,  typing,  moving thumb over surface | moving index up- and downwards,  pushing piano key,  typing,  moving index over surface | moving little finger up- and downwards,  pushing piano key,  typing,  moving little finger over surface |
| 7 | pre | playing musical instrument,  scratching over table,  moving up and down in the air | playing musical instrument,  scratching over table,  moving up and down in the air | playing musical instrument,  scratching over table,  moving up and down in the air |
|  | post | moving it in a round circle,  cutting the cake with one finger | moving it in a round circle,  cutting the cake with one finger | moving it in a round circle,  cutting the cake with one finger |
| 8 | pre | moving finger to the left and right,  spreading | typing | moving finger to the left and right,  spreading |
|  | post | bending | typing | spreading |
| 9 | pre | kneading bread,  testing if it is ready to bake,  making circles | looking through a map,  pointing at landmark/location,  making circles | making circles,  imagining the circles and other fingers in ice |
|  | post | pressing my side,  making circles,  testing bread dough | looking through a map,  pointing at landmark,  making circles and lifting | lifting the little finger |
| 10 | pre | counting number of guests with counting device,  welcoming passengers | set timer on the oven | Groove of MRI |
|  | post | home button on phone,  counting number of guests | set timer on the oven | typing on the edge of MRI |
| 11 | pre | pressing a button | pressing a button | moving finger up and down |
|  | post | pressing a button | pressing a button | moving finger up and down  pressing a button (as alternative) |
| 12 | pre | pressing on surface | pressing on surface | stretching outwards |
|  | post | pressing outwards | pressing on surface | stretching outwards |
| 13 | pre | thumb game,  twiddling the thumbs,  à in sign language | ‘worm song’ with finger,  making circles with index finger | ‘worm song’ with finger,  making circles with little finger |
|  | post | à in sign language | ‘worm song’ | ‘worm song’ |
| 14 | pre | thumb war,  pressing controller button | pressing controller button,  rotating a ring around using index finger,  tapping on the desk | pinky swear,  moving little finger around into a water bowl |
|  | post | extending my thumb  thumb war,  tapping on smartphone | sliding finger on surface, especially paper | pinky swear |
| 15 | pre | rotating controller of a beamer,  movements of pressing, extension, adduction, abduction and rotation | rotating controller of a beamer,  movements of pressing, extension, adduction, abduction and rotation | movements of extension, adduction, abduction, rotation, pulling a hook downwards |
|  | post | rotating controller of a beamer,  movements of pressing, extension, adduction, abduction and rotation,  pressing spacebar on keyboard,  using gaming controller | rotating controller of a beamer,  movements of pressing, extension, adduction, abduction and rotation,  pressing piano key and keyboard | movements of extension, adduction, abduction, rotation, pulling a hook downwards,  pressing piano key |
| 16 | pre | making circles (like holding a joystick),  up- and downwards movements,  grabbing a handle | pulling a trigger,  picking nose,  scratching the head | moving finger left, right, up, down, pushing outwards,  holding a rope, |
|  | post | moving finger in figure of 8, up and down,  apply sunscreen | Tickling,  swiping a screen,  moving finger in figure of 8 | moving finger to the side,  swipe inside of a ham jar |
