# Supplementary material for: TMS-Based Neurofeedback Training of Mental Finger Individuation Induces Neuroplastic Changes in the Sensorimotor System
Source: J Neurosci. 2025 Jul 24;45(35):e2189242025. doi: 10.1523/JNEUROSCI.2189-24.2025 (PMC12392065; doi:10.1523/JNEUROSCI.2189-24.2025)
Supplement: Figure 4-1 — Number of repetitions for the paired-pulse TMS protocols in the pre- and post-training TMS sessions. Download Figure 4-1, DOCX file. [file jneuro-45-e2189242025-s004.docx]

| **NF group** | **Session** | **SICI** | **ICF** | **Single pulse** |
| --- | --- | --- | --- | --- |
| 1 | pre | 1 | 1 | 1 |
|  | post | 1 | 2 | 1 |
| 2 | pre | 2 | 1 | 1 |
|  | post | 1 | 1 | 2 |
| 3 | pre | 1 | 1 | 2 |
|  | post | 1 | 3 | 1 |
| 4 | pre | 1 | 1 | 2 |
|  | post | 2 | 1 | 2 |
| 5 | pre | 2 | 1 | 2 |
|  | post | 1 | 1 | 2 |
| 6 | pre | 2 | 1 | 2 |
|  | post | 1 | 1 | 2 |
| 7 | pre | 2 | 1 | 1 |
|  | post | 2 | 1 | 2 |
| 8 | pre | 2 | 1 | 2 |
|  | post | 1 | 1 | 2 |
| 9 | pre | 2 | 1 | 1 |
|  | post | 1 | 2 | 1 |
| 10 | pre | 2 | 1 | 1 |
|  | post | 1 | 1 | 2 |
| 11 | pre | 1 | 1 | 2 |
|  | post | 2 | 1 | 1 |
| 12 | pre | 2 | 2 | 1 |
|  | post | 2 | 1 | 1 |
| 13 | pre | 1 | 1 | 2 |
|  | post | 1 | 1 | 2 |
| 14 | pre | 1 | 2 | 2 |
|  | post | 2 | 1 | 1 |
| 15 | pre | 2 | 1 | 2 |
|  | post | 1 | 2 | 1 |
| 16 | pre | 1 | 2 | 2 |
|  | post | 2 | 1 | 1 |
|  | | | | |
| **Control group** | **Session** | **SICI** | **ICF** | **Single pulse** |
| 1 | pre | 1 | 1 | 2 |
|  | post | 1 | 2 | 1 |
| 2 | pre | 2 | 1 | 2 |
|  | post | 2 | 2 | 2 |
| 3 | pre | 1 | 1 | 2 |
|  | post | 1 | 1 | 2 |
| 4 | pre | 2 | 1 | 1 |
|  | post | 1 | 1 | 2 |
| 5 | pre | 2 | 1 | 2 |
|  | post | 1 | 1 | 1 |
| 6 | pre | 1 | 2 | 2 |
|  | post | 1 | 1 | 2 |
| 7 | pre | 1 | 1 | 2 |
|  | post | 1 | 2 | 1 |
| 8 | pre | 1 | 1 | 1 |
|  | post | 1 | 1 | 2 |
| 9 | pre | 1 | 2 | 2 |
|  | post | 2 | 1 | 1 |
| 10 | pre | 1 | 1 | 2 |
|  | post | 1 | 1 | 2 |
| 11 | pre | 1 | 1 | 2 |
|  | post | 2 | 1 | 1 |
| 12 | pre | 1 | 2 | 2 |
|  | post | 1 | 1 | 2 |
| 13 | pre | 1 | 2 | 1 |
|  | post | 1 | 2 | 2 |
| 14 | pre | 2 | 1 | 1 |
|  | post | 1 | 2 | 1 |
| 15 | pre | 2 | 1 | 1 |
|  | post | 1 | 1 | 2 |
| 16 | pre | 2 | 1 | 1 |
|  | post | 1 | 2 | 1 |
