# Supplementary material for: TMS-Based Neurofeedback Training of Mental Finger Individuation Induces Neuroplastic Changes in the Sensorimotor System
Source: J Neurosci. 2025 Jul 24;45(35):e2189242025. doi: 10.1523/JNEUROSCI.2189-24.2025 (PMC12392065; doi:10.1523/JNEUROSCI.2189-24.2025)
Supplement: Figure 5-1 — Motor imagery network. Activation clusters, corresponding size, anatomical region, FW-corrected p-value for multiple comparisons, peak coordinate in MNI space, and maximum z-value of the reported contrast of motor imagery (pre-training session, across all fingers and groups) vs rest thresholded at Z > 3.1. Reported anatomical labels were determined using the Jülich Histological (Eickhoff et al., 2005), the Harvard-Oxford cortical (Desikan et al., 2006) and subcortical structural (Frazier et al., 2005), and the probabilistic cerebellar atlases (Diedrichsen et al., 2009), correspond to the location of maxima within each cluster. Download Figure 5-1, DOCX file. [file jneuro-45-e2189242025-s005.docx]

| **Both groups, pre-training session: Motor imagery > rest** | | | | | | | |
| --- | --- | --- | --- | --- | --- | --- | --- |
| Cluster | # voxels | Region of peak | *p*_(FWE)_ | Peak coordinates | | | z-value |
|  |  |  |  | X | Y | Z |  |
| 1 | 6300 | Left premotor cortex | 0 | -56 | 6 | 20 | 6.5 |
| 2 | 1947 | Left inferior parietal lobe | 1.4e-42 | -50 | -28 | 36 | 5.99 |
| 3 | 1161 | Right premotor cortex | 8.24e-30 | 56 | 6 | 18 | 5.77 |
| 4 | 922 | Right VI | 2.17e-25 | 34 | -58 | -24 | 6.26 |
| 5 | 649 | Right VIIIa | 7.77e-20 | 28 | -60 | -54 | 6.54 |
| 6 | 134 | Left visual cortex V2 | 3.93e-06 | -12 | -92 | -4 | 5.21 |
| 7 | 129 | Right visual cortex V1 | 5.96e-06 | 14 | -90 | 0 | 4.7 |
| 8 | 115 | Right premotor cortex | 2.02e-05 | 26 | -12 | 50 | 4.15 |
| 9 | 103 | Left middle frontal gyrus | 5.97e-05 | -40 | 38 | 28 | 4.17 |
| 10 | 95 | Left VI | 0.000126 | -30 | -54 | -28 | 4.53 |
| 11 | 85 | Right hippocampus | 0.00033 | 20 | -14 | -18 | 5.31 |
| 12 | 72 | Left VIIIa | 0.00122 | -30 | -58 | -56 | 5 |
| 13 | 68 | Left Crus I | 0.00186 | -44 | -56 | -30 | 5.41 |
| 14 | 63 | Left hippocampus | 0.00317 | -20 | -16 | -14 | 5.2 |
| 15 | 46 | Left thalamus | 0.0218 | -8 | -20 | -2 | 5.43 |
| 16 | 40 | Right anterior intra-parietal sulcus/ superior parietal lobule | 0.045 | 36 | -34 | 40 | 3.89 |
