# Supplementary material for: TMS-Based Neurofeedback Training of Mental Finger Individuation Induces Neuroplastic Changes in the Sensorimotor System
Source: J Neurosci. 2025 Jul 24;45(35):e2189242025. doi: 10.1523/JNEUROSCI.2189-24.2025 (PMC12392065; doi:10.1523/JNEUROSCI.2189-24.2025)
Supplement: Figure 5-2 — Pre- to post-training changes. Activation clusters, corresponding size, anatomical region, FW-corrected p-value for multiple comparisons, peak coordinate in MNI space, and maximum z-value of the reported pre- to post-training contrasts thresholded at Z > 3.1. Reported anatomical labels were determined using the Jülich Histological (Eickhoff et al., 2005), the Harvard-Oxford cortical (Desikan et al., 2006) and subcortical structural (Frazier et al., 2005), and the probabilistic cerebellar atlases (Diedrichsen et al., 2009), correspond to the location of maxima within each cluster. Download Figure 5-2, DOCX file. [file jneuro-45-e2189242025-s006.docx]

| **NF group: Pre-training > Post-training**  None  **NF group: Post-training > Pre-training** | | | | | | | |
| --- | --- | --- | --- | --- | --- | --- | --- |
| Cluster | # voxels | Region of peak | *p*_(FWE)_ | Peak coordinates | | | z-value |
|  |  |  |  | X | Y | Z |  |
| 1 | 61 | Right precuneous | 0.00115 | 10 | -70 | 36 | 3.76 |
| **Control group: Pre-training > Post-training**  None  **Control group: Post-training > Pre-training** | | | | | | | |
| 1 | 380 | Right inferior parietal lobule | 9.96e-17 | 56 | -44 | 26 | 4.33 |
| 2 | 190 | Right anterior intra-parietal sulcus | 4.92e-10 | 38 | -52 | 38 | 4.23 |
| 3 | 162 | Right middle temporal gyrus | 6.94e-09 | 60 | -20 | -10 | 4.15 |
| 4 | 107 | Bilateral posterior cingulate gyrus / precuneous | 2.09e-06 | 2 | -48 | 36 | 3.88 |
| 5 | 70 | Right middle frontal gyrus | 0.00017 | 28 | 18 | 54 | 3.92 |
| 6 | 63 | Left superior lateral occipital cortex | 0.000425 | -30 | -74 | 26 | 4.43 |
| 7 | 61 | Left Crus I | 0.000556 | -12 | -76 | -30 | 4.12 |
| 8 | 42 | Right superior parietal lobule | 0.00832 | 4 | -38 | 48 | 4.32 |
| 9 | 41 | Right middle frontal gyrus | 0.00969 | 50 | 20 | 30 | 3.68 |
