# Supplementary material for: TMS-Based Neurofeedback Training of Mental Finger Individuation Induces Neuroplastic Changes in the Sensorimotor System
Source: J Neurosci. 2025 Jul 24;45(35):e2189242025. doi: 10.1523/JNEUROSCI.2189-24.2025 (PMC12392065; doi:10.1523/JNEUROSCI.2189-24.2025)
Supplement: Figure 5-3 — Interaction analysis. Activation clusters, corresponding size, anatomical region, FW-corrected p-value for multiple comparisons, peak coordinate in MNI space, and maximum z-value of the reported interaction analysis where the relationship between pre- to post-training changes and motor imagery performance changes differs between the NF and control groups, thresholded at Z > 3.1. Reported anatomical labels were determined using the Jülich Histological (Eickhoff et al., 2005), the Harvard-Oxford cortical (Desikan et al., 2006) and subcortical structural (Frazier et al., 2005), and the probabilistic cerebellar atlases (Diedrichsen et al., 2009), correspond to the location of maxima within each cluster. Download Figure 5-3, DOCX file. [file jneuro-45-e2189242025-s007.docx]

| **Interaction effect of Group and Covariate: NF group > Control group** | | | | | | | |
| --- | --- | --- | --- | --- | --- | --- | --- |
| Cluster | # voxels | Region of peak | *p*_(FWE)_ | Peak coordinates | | | z-value |
|  |  |  |  | X | Y | Z |  |
| 1 | 229 | Left primary motor cortex | 7.59e-11 | -34 | -28 | 58 | 5.01 |
| 2 | 132 | Left frontal pole | 4.17e-07 | -34 | 52 | 4 | 4.08 |
| 3 | 128 | Right V | 5.96e-07 | 20 | -44 | -20 | 4.62 |
| 4 | 128 | Right paracingulate gyrus | 5.69e-07 | 12 | 14 | 38 | 4.59 |
| 5 | 100 | Right SMA | 1.1e-05 | -4 | 2 | 60 | 4.69 |
| 6 | 70 | Right VIIIa | 0.000331 | 14 | -68 | -48 | 4.58 |
| 7 | 66 | Left temporal pole | 0.00054 | -50 | 16 | -8 | 4.55 |
| 8 | 60 | Left premotor cortex | 0.00115 | -52 | -2 | 34 | 4.14 |
| 9 | 53 | Left precuneous | 0.00284 | -8 | -72 | 28 | 4.12 |
| 10 | 45 | Left primary somatosensory cortex | 0.00844 | -50 | -36 | 56 | 4.12 |
| 11 | 38 | Left precuneous | 0.023 | -8 | -72 | 46 | 4.6 |
| 12 | 37 | Left superior parietal lobule | 0.0266 | -22 | -66 | 54 | 4 |
| 13 | 33 | Left premotor cortex | 0.0484 | -26 | -6 | 50 | 3.87 |
| **Interaction effect of Group and Covariate: Control group > NF group** | | | | | | | |
| 1 | 37 | Left middle temporal gyrus | 0.0266 | -64 | -6 | -28 | 4.48 |
